# *In*-*vivo* correlations between skin metabolic oscillations and vasomotion in wild-type mice and in a model of oxidative stress

**DOI:** 10.1038/s41598-018-36970-4

**Published:** 2019-01-17

**Authors:** Salvatore Smirni, Alison D. McNeilly, Michael P. MacDonald, Rory J. McCrimmon, Faisel Khan

**Affiliations:** 1 University of Dundee, School of Medicine, Dundee, Scotland UK; 2 University of Dundee, School of Science and Engineering, Dundee, Scotland UK

## Abstract

Arterioles in the cutaneous microcirculation frequently display an oscillatory phenomenon defined vasomotion, consistent with periodic diameter variations in the micro-vessels associated with particular physiological or abnormal conditions. The cellular mechanisms underlying vasomotion and its physiological role have not been completely elucidated. Various mechanisms were demonstrated, based on cell Ca^2+^ oscillations determined by the activity of channels in the plasma membrane or sarcoplasmic reticulum of vascular cells. However, the possible engagement in vasomotion of cell metabolic oscillations of mitochondrial or glycolytic origin has been poorly explored. Metabolic oscillations associated with the production of ATP energy were previously described in cells, while limited studies have investigated these fluctuations *in*-*vivo*. Here, we characterised a low-frequency metabolic oscillator (MO-1) in skin from live wild-type and Nrf2^−/−^ mice, by combination of fluorescence spectroscopy and wavelet transform processing technique. Furthermore, the relationships between metabolic and microvascular oscillators were examined during phenylephrine-induced vasoconstriction. We found a significant interaction between MO-1 and the endothelial EDHF vasomotor mechanism that was reduced in the presence of oxidative stress (Nrf2^−/−^ mice). Our findings suggest indirectly that metabolic oscillations may be involved in the mechanisms underlying endothelium-mediated skin vasomotion, which might be altered in the presence of metabolic disturbance.

## Introduction

The term vasomotion indicates rhythmic oscillations of blood vessels diameter responsible for changes in the vascular tone and blood perfusion to tissue^1,2^. The physiological role of these oscillations is unclear, with contrasting studies supporting the development of vasomotion in both diseased and healthy conditions^2^. The origin of the oscillations is mainly determined by the local contraction and relaxation of vascular smooth muscle cells (VSMCs), which act as synchronised pacemakers in the vessels wall^1–3^. Although in several arteries (e.g. rat aorta) the oscillations may depend exclusively on the activity of VSMCs^1^, in other vascular beds (i.e. rat mesenteric artery) the most internal layer of blood vessels (endothelium) plays an important modulatory role during arterial motion^4,5^. Vasomotion is more prominent in small resistance micro-vessels^2^ such as skin arterioles that represent an optimal sample for the *in*-*vivo* examination of this phenomenon reflecting the general health conditions of the cardiovascular system^6^.

Non-invasive observation of skin vasomotion can be achieved by direct methods measuring vessels diameter, e.g. intravital video-microscopy^3,4^, or by indirect techniques such as Laser Doppler Flowmetry (LDF) based on monitoring the heterogeneous fluctuations of microvascular blood perfusion signal^2^. The combination of LDF with the wavelet transform signal processing analysis has revealed oscillations in the human cutaneous microcirculation that relate to physiological phenomena: myogenic (52–145 × 10^−3^ Hz), neurogenic (21–52 × 10^−3^ Hz), endothelial nitric oxide (NO)-dependent (9.5–21 × 10^−3^ Hz), and endothelial NO-independent (5–9.5 × 10^−3^ Hz)^7,8^.

For the explanation of the cellular mechanisms causing the oscillations of vessels diameter observed during vasomotion, three different kinds of cellular oscillators have been proposed as possible drivers of this phenomenon: cytosolic oscillator, membrane oscillator and metabolic oscillator^1,2^. The importance of the cooperation between cytosolic and membrane oscillators to enhance the VSMCs synchronisation responsible for vasomotion has been largely explored and recognised^1,2,9–12^. They act inducing Ca^2+^ fluctuations in VSMCs, respectively through the oscillatory release of Ca^2+^ from the sarcoplasmic reticulum^1,10,11^, and via the opening activity of voltage-dependent Ca^2+^ channels and large-conductance K^+^ channels of the plasma membrane^1,2,12^. Furthermore, Ca^2+^ and membrane potential oscillations have been described in endothelial cells (ECs), suggesting that in some vascular beds (i.e. rat mesenteric artery) in response to specific stimuli (e.g. *α*-adrenergic stimulation) a primary oscillation from ECs and the interaction with VSMCs might be essential for vasomotion^13–15^.

A third hypothesised driving mechanism is the presence of a metabolic oscillator represented by oscillations in the activity of the glycolytic enzyme phosphofructokinase (PFK) that cause fluctuations in glycolysis, ATP levels and in the activity of plasma membrane ion channels and membrane potential^1^. This mechanism has never been considered important because of limited experimental evidence for the involvement of PFK in glycolytic and Ca^2+^ fluctuations^1,2^. However, recent studies have strongly suggested the participation of PFK in reactions responsible for glycolysis and Ca^2+^ oscillations^16–18^, with a possible driving effect exerted *in*-*vivo* by external glucose availability and uptake^18–20^. Furthermore, metabolic mitochondrial oscillations have also been reported, mainly associated with fluctuations of the mitochondrial membrane potential ΔΨm that may be mediated by calcium^21–23^. The external oxygen required for sustaining the electron transport chain has been proposed as the driving force for the mitochondrial oscillator^18^. All this evidence suggests that the energetic and oxygen tissue requirements might stimulate vasomotion phenomena associated with Ca^2+^ oscillations of metabolic origin, which may involve the intercellular communication between heterogeneous groups of cells (e.g. ECs, VSMCs and cells of the tissue surrounding blood vessels).

Metabolic oscillations have been studied in different cell types, i.e. yeast *Saccharomyces cerevisiae*^19,21^, cardiac myocytes^24^, *β*-cells^23^, and can be detected indirectly by measuring the fluctuations in the autofluorescence of the intermediate product of energy metabolism NAD(P)H (nicotamide adenine dinucleotide). However, there are limited studies describing metabolic oscillations in live tissue. Mitochondrial fast oscillations have been reported *in*-*vivo* in rat, and detected by intravital two-photon (2P) microscopy measurements of NAD(P)H^25^. Nevertheless, to our knowledge, the slow metabolic NAD(P)H oscillations described in many *in*-*vitro* or *ex*-*vivo* cell studies^16,23^ have not been investigated *in*-*vivo*.

In the present study, we have utilised skin tissue from live mice to simultaneously measure NAD(P)H and microvascular blood flow respectively by laser fluorescence spectroscopy (LFS) and LDF, under physiological conditions and during *α*-adrenergic stimulation with the vasoconstrictor drug phenylephrine (PE). Data were processed by continuous wavelet transform (CWT) spectral analysis to characterise heterogeneous oscillations of NAD(P)H and LDF signals. We found low-frequency oscillations of NAD(P)H autofluorescence with periods of 2.4 min, 5 min and 10 min. To our knowledge, this is the first study in live skin tissue of slow NAD(P)H oscillations previously described in cells. Furthermore, we found relevant correlations between metabolic and vasomotion oscillations suggesting a link between these processes. Therefore, our results support the idea of vasomotion as a dynamic process in the context of a metabolically active microvascular network involving the cooperation of different cell types and vascular segments^2,15^, and a possible driving mechanism of metabolic origin.

## Results

### Nrf2^−/−^ (Nuclear factor-erythroid 2 p45-related factor 2) knockout model

**Figure 1:**
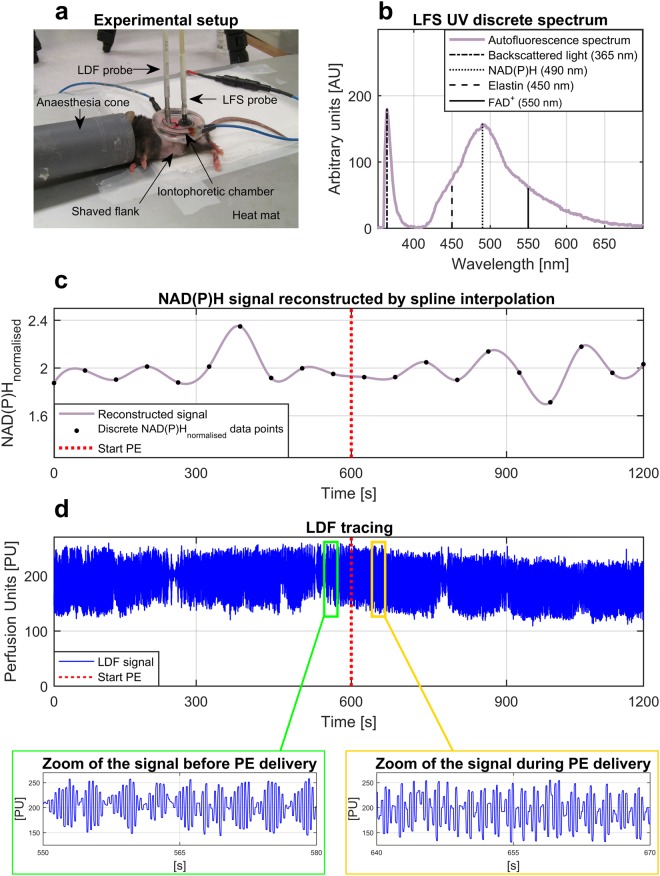
Example of data collection from mouse skin. (**a**) Experimental setup for LFS and LDF recordings during local transdermal administration of 1% phenylephrine by iontophoresis. (**b**) Example of single UV discrete autofluorescence spectrum measured by LFS. (**c**) Example of 20 min NAD(P)H signal reconstructed by piecewise cubic spline interpolation of NAD(P)H_*normalised*_ values extracted from 10 baseline UV spectra and 10 UV spectra collected during PE administration. (**d**) Example of 20 min blood flow tracing measured by LDF during iontophoresis.

Experiments were performed on 5 wild-type (WT) control and 6 Nrf2^−/−^ mice. The Nrf2 transcription factor is a key regulator of the cell redox state, involved in the resistance to oxidative stress by activating the cellular antioxidant defence^26^. Nrf2 knockout leads to abnormal mitochondrial activity, altered redox status, increased oxidative stress, and has an impact on cardiovascular dynamics^26,27^. We used the Nrf2^−/−^ strain to investigate the differences in metabolic and vascular dynamics between normal mice and animals with impaired antioxidant defence that may favour the development of metabolic and cardiovascular disorders. Figure 1 shows the experimental setup (Fig. 1a), and examples of simultaneous LFS and LDF signals (Fig. 1c,d) from the flank of a mouse at baseline (10 min) and during vasoconstriction induced by local administration of PE (10 min). The reason for using PE is that, according to Okazaki *et al*.^5^, this vasoactive agent is able to stimulate a vasomotion phenomenon mediated by endothelial cells. Thus, the use of PE provided the opportunity to study at the same time the role of the endothelium in vasomotion and its relationship with metabolic oscillations. Because of the small number of animals available in the study, the experiments were repeated in duplicate for each mouse and the average value of each variable between the two experiments was taken as final value for data analysis. Data of all the variables analysed in this study are summarised in Table 1.

**Table 1:** Average values of all the variables included in the study.

Parameters	WT (Mean ± SD)		Nrf2^−/−^ (Mean ± SD)	
Variable name	Baseline	Phenylephrine	Baseline	Phenylephrine
Blood perfusion [PU]	174.0 ± 30.40	162.8 ± 26.80	197.6 ± 20.50	184.0 ± 16.70
SO_2_ [%]	47.3 ± 9.60	44.9 ± 10.7	48.3 ± 9.60	45.5 ± 6.10
NAD(P)H_*normalised*_	1.7 ± 0.2	1.7 ± 0.2	1.9 ± 0.2	1.8 ± 0.1
RR_*index*_	2.2 ± 0.1	2.2 ± 0.1	2.0 ± 0.1	2.0 ± 0.1
*e*_*i* *Endothelial* *EDHF*_	(0.70 ± 0.20) × 10^−3^	(0.40 ± 0.10) × 10^−3^	(0.40 ± 0.20) × 10^−3^	(0.60 ± 0.30) × 10^−3^
*e*_*i* *Endothelial* *NO*_	(1.10 ± 0.30) × 10^−3^	(0.60 ± 0.20) × 10^−3^	(0.80 ± 0.30) × 10^−3^	(1.00 ± 0.50) × 10^−3^
*e*_*i* *Neurogenic*_	(1.90 ± 0.80) × 10^−3^	(1.00 ± 0.30) × 10^−3^	(1.30 ± 0.40) × 10^−3^	(1.50 ± 0.80) × 10^−3^
*e*_*i* *Myogenic*_	(4.50 ± 1.50) × 10^−3^	(3.20 ± 1.10) × 10^−3^	(3.40 ± 0.60) × 10^−3^	(3.80 ± 1.10) × 10^−3^
*a*_*i* *Endothelial* *EDHF*_	(11.0 ± 2.30) × 10^−3^	(6.10 ± 1.50) × 10^−3^	(7.10 ± 4.10) × 10^−3^	(8.90 ± 4.00) × 10^−3^
*a*_*i* *Endothelial* *NO*_	(10.6 ± 4.40) × 10^−3^	(6.00 ± 2.60) × 10^−3^	(7.60 ± 3.90) × 10^−3^	(9.50 ± 5.60) × 10^−3^
*a*_*i* *Neurogenic*_	(12.9 ± 7.60) × 10^−3^	(5.50 ± 1.80) × 10^−3^	(8.40 ± 1.90) × 10^−3^	(8.40 ± 4.70) × 10^−3^
*a*_*i* *Myogenic*_	(14.8 ± 6.60) × 10^−3^	(9.70 ± 4.30) × 10^−3^	(11.2 ± 2.40) × 10^−3^	(12.4 ± 5.30) × 10^−3^
*f*_*Endothelial* *EDHF*_ [Hz]	(8.00 ± 0.60) × 10^−3^	(6.90 ± 0.90) × 10^−3^	(6.30 ± 1.00) × 10^−3^	(7.70 ± 0.80) × 10^−3^
*f*_*Endothelial* *NO*_ [Hz]	(1.40 ± 0.10) × 10^−2^	(1.50 ± 0.30) × 10^−2^	(1.40 ± 0.20) × 10^−2^	(1.50 ± 0.20) × 10^−2^
*f*_*Neurogenic*_ [Hz]	(2.80 ± 0.50) × 10^−2^	(3.00 ± 0.70) × 10^−2^	(3.50 ± 1.00) × 10^−2^	(4.00 ± 1.00) × 10^−2^
*f*_*Myogenic*_ [Hz]	(10.4 ± 1.30) × 10^−2^	(9.30 ± 2.10) × 10^−2^	(9.20 ± 1.70) × 10^−2^	(9.20 ± 1.60) × 10^−2^
*e*_*i* *NAD*( *P*) *H* *MO*–1_	(12.8 ± 1.40) × 10^−3^	(16.1 ± 1.50) × 10^−3^	(12.3 ± 4.60) × 10^−3^	(15.8 ± 2.40) × 10^−3^
*e*_*i* *RR* *MO*–1_	(11.7 ± 4.60) × 10^−3^	(14.9 ± 2.50) × 10^−3^	(14.6 ± 3.90) × 10^−3^	(12.0 ± 3.10) × 10^−3^
*a*_*i* *NAD*( *P*) *H* *MO*–1_	(10.7 ± 2.70) × 10^−3^	(11.7 ± 3.50) × 10^−3^	(9.60 ± 5.40) × 10^−3^	(13.8 ± 5.50) × 10^−3^
*a*_*i* *RR* *MO*–1_	(8.10 ± 4.40) × 10^−3^	(12.4 ± 2.30) × 10^−3^	(11.2 ± 3.90) × 10^−3^	(8.60 ± 4.20) × 10^−3^
*f*_*NAD*(*P*)*H* *MO*–1_ [Hz]	(6.10 ± 1.10) × 10^−3^	(6.60 ± 0.60) × 10^−3^	(6.80 ± 1.40) × 10^−3^	(6.30 ± 0.90) × 10^−3^
*f*_*RR* *MO*–1_ [Hz]	(6.70 ± 1.10) × 10^−3^	(6.40 ± 1.20) × 10^−3^	(6.30 ± 0.80) × 10^−3^	(7.00 ± 1.30) × 10^−3^
C*ϕ*_*NAD*(*P*)*H* *MO*–1/*EDHF*_	0.78 ± 0.03	0.76 ± 0.05	0.76 ± 0.05	0.79 ± 0.05
C*ϕ*_*RR* *MO*–1/*EDHF*_	0.79 ± 0.04	0.76 ± 0.02	0.74 ± 0.06	0.78 ± 0.02

Data are presented as mean ± Standard Deviation (SD). PU = Perfusion arbitrary units. SO_2_ = Oxygen saturation (%). NAD(P)H_*normalised*_ = Autofluorescence of the reduced nicotamide adenine dinucleotide coenzyme, normalised according to equation 2 (dimensionless). RR_*index*_ = Redox ratio index obtained according to equation 1 (dimensionless). *e*_*i*_ = Relative energy of wavelet oscillators (dimensionless). *a*_*i*_ = Relative amplitude of wavelet oscillators (dimensionless). *f* = frequency of wavelet oscillators (Hz). LDF oscillators: endothelial EDHF, endothelial NO, neurogenic, myogenic. Metabolic oscillators: NAD(P)H MO-1, RR MO-1. C*ϕ*_*NAD*(*P*)*H* *MO*–1/*EDHF*_ = Phase coherence (dimensionless) between NAD(P)H MO-1 and endothelial EDHF oscillators. C*ϕ*_*RR* *MO*–1/*EDHF*_ = Phase coherence (dimensionless) between RR MO-1 and endothelial EDHF oscillators.

### General microvascular and metabolic biomarkers

**Figure 2:**
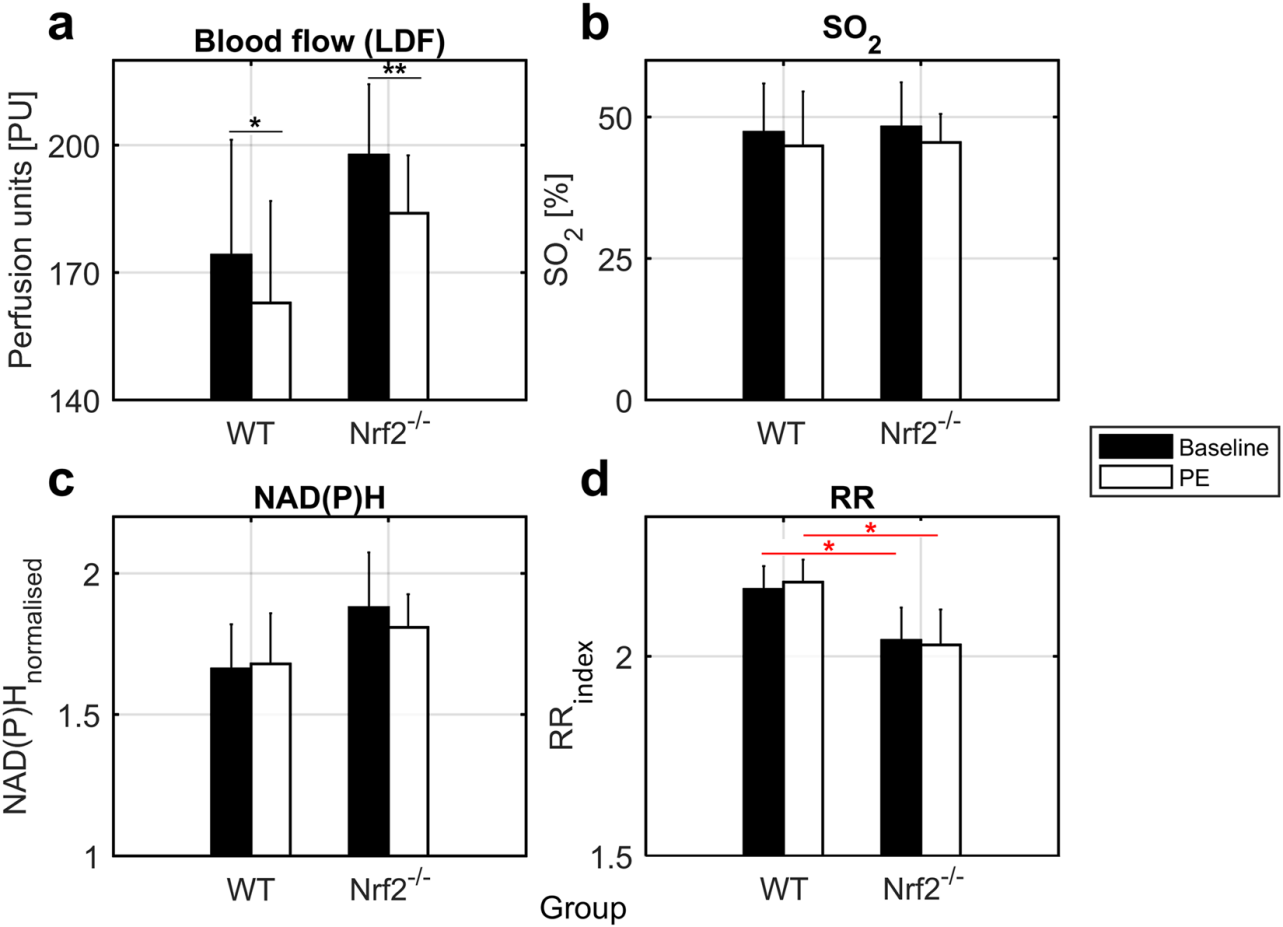
Average trends of microvascular and metabolic biomarkers. (**a**) Blood flow (PU). (**b**) SO_2_ (%). (**c**) NAD(P)H_*normalised*_ (dimensionless). (**d**) RR_*index*_ (dimensionless). Error bars = 2 standard errors (SE). Black bars = Baseline. White bars = PE. WT = Control. Nrf2^−/−^ = Knockout. Black lines/asterisks = Significant changes during PE delivery. Red lines/asterisks = Significant differences between groups. t-test *p < 0.05, **p < 0.01.

Results of blood perfusion and oxygen saturation (SO_2_) are shown in Fig. 2a,b. Blood perfusion decreased to a similar degree in WT (p = 0.046) and Nrf2^−/−^ (p = 0.007) mice during PE-induced vasoconstriction, however, a stronger p-value was found in knockout mice. SO_2_, measured by reflectance spectroscopy (RS), was similar between groups and decreased slightly following vasoconstriction without statistical significance.

LFS skin irradiation by UV light stimulates the autofluorescence emission of multiple fluorophores, including NAD(P)H (490 nm), flavin adenine dinucleotide (FAD^+^, 550 nm) and elastin (450 nm) (Fig. 1b). Although the NAD(P)H central emission peak represents the highest contribution to the spectrum, we took advantage of the overlapping contributions from FAD^+^ and elastin, respectively to estimate the redox ratio (RR) index and to normalise NAD(P)H fluorescence:

1 $$R{R}_{index}=\frac{NAD(P){H}_{amplitude}}{FA{D}_{amplitude}^{+}}.$$

2 $$NAD(P){H}_{normalised}=\frac{NAD(P){H}_{amplitude}}{Elasti{n}_{amplitude}}.$$

RR_*index*_ is a measure of the mitochondrial redox state, reflecting the balance between the reduced NAD(P)H and the oxidised FAD^+^ coenzymes. Comparing this index between WT and Nrf2^−/−^ mice should provide discriminatory data, confirming that the Nrf2^−/−^ phenotype is characterised by altered mitochondrial function due to impaired antioxidant defence and higher oxidative stress. NAD(P)H autofluorescence was normalised by elastin’s fluorescence peak to reduce blood volume artefacts (see more details in the Supplementary Information).

During each experiment, 20 UV spectra were collected in 20 min at a rate of a spectrum every minute, 10 at baseline and 10 during PE administration. RR_*index*_ and NAD(P)H_*normalised*_ values were estimated from each spectrum and used to reconstruct a time series (Fig. 1c) for applying the CWT spectral analysis. Figure 2c,d describe the average trends of NAD(P)H_*normalised*_ and RR_*index*_ signals. NAD(P)H_*normalised*_ did not show significant statistical differences between groups, while RR_*index*_ was significantly lower in Nrf2^−/−^ mice (p = 0.016) confirming that knockout animals were affected by altered redox state and higher oxidative stress.

### Reproduction of the LDF oscillators reported in the literature

**Figure 3:**
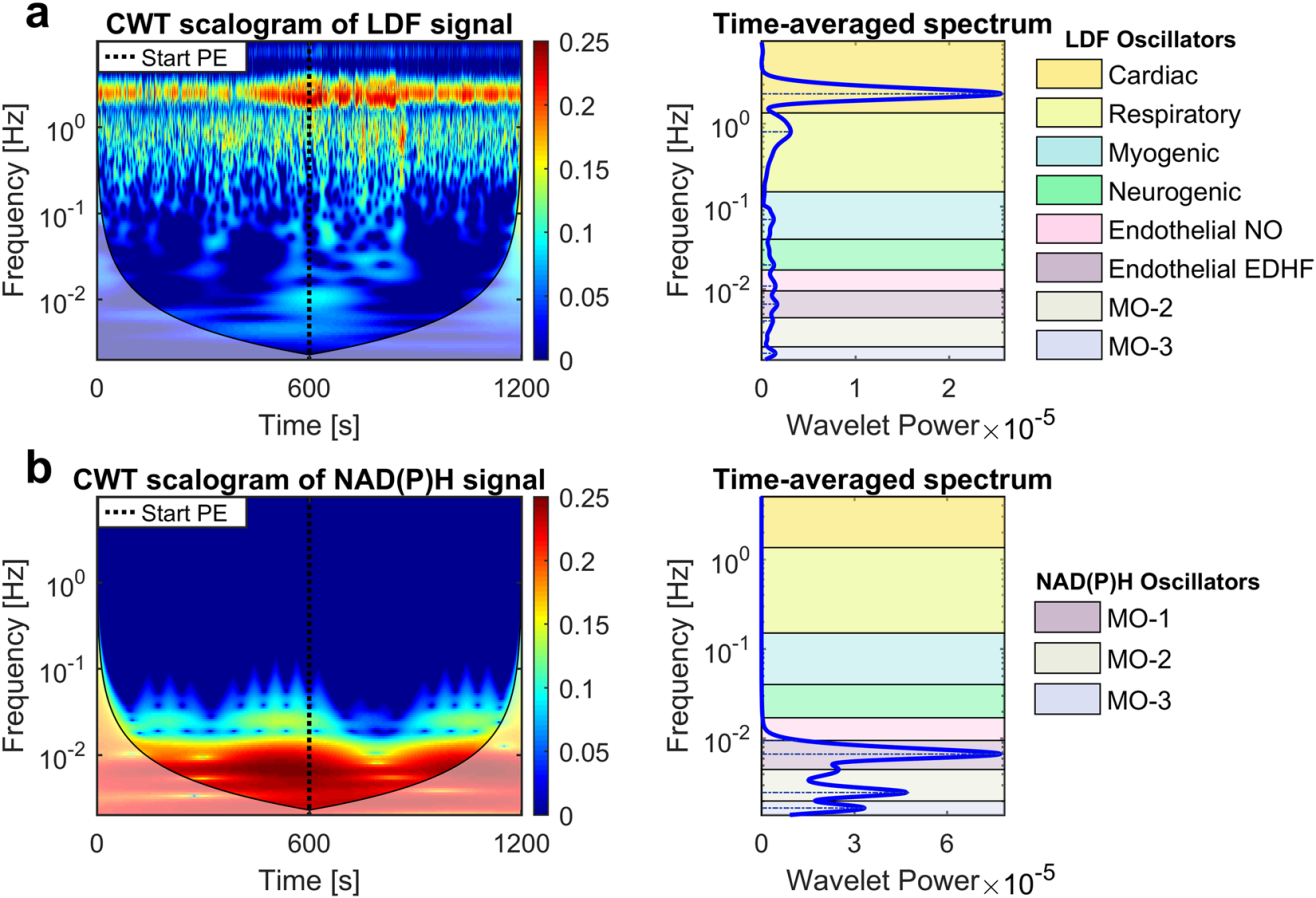
CWT data processing of LDF and NAD(P)H signals. (**a**) Example of CWT scalogram (left) and corresponding time-averaged spectrum (right) from LDF signal. The scalogram describes the distribution of the wavelet spectral power/energy in the time-frequency domain using a gradient coloured map ranging from dark blue (low energy) to dark red (high energy). The chart displays a high-energy continuous band in the cardiac frequency interval, confirming that data were collected correctly. Physiologically, the wavelet energy represents a measure of how much a physiological component defined by a specific frequency interval contributes to blood flow signal at a specific time. The two transparent regions at the bottom-right and bottom-left of the scalogram represent regions outside of the “cone of influence” where data might not be reliable (frequencies < 5 × 10^−3^ Hz). The cone of influence is a time-frequency region where distortions of the CWT due to the finite temporal period of the measured signal are not relevant^34^. Instead, the areas outside of the cone are close to the time limits of the time series, where the wavelet transform is affected by boundary effects making the calculations from this time-frequency region imprecise^34^. The time-averaged spectrum (right graph) allows discriminating the wavelet energy peaks of the different oscillators at specific frequency intervals. As shown by the coloured tags in the legend, we identified the typical oscillators reported in literature^8^: (I) Cardiac, (II) Respiratory, (III) Myogenic, (IV) Neurogenic, (V) Endothelial NO-dependent, (VI) Endothelial NO-independent (EDHF). Comparing the wavelet components between mice groups is powerful to distinguish healthy and diseased vascular conditions by quantifying the contribution of specific biological components. (**b**) Example of CWT analysis of the NAD(P)H_*normalised*_ reconstructed signal. Three low-frequency metabolic oscillators were characterised: Metabolic oscillator-1 (MO-1), MO-2 and MO-3. These oscillators might reflect specific dynamic patterns of ATP energy production in the cutaneous tissue, which may be variable depending on the presence of healthy or diseased conditions. In this study, we focused on the dynamics of MO-1 because MO-2 and MO-3 intervals are located outside of the cone of influence where data might be inaccurate.

Vasomotion oscillations were assessed by combining LDF and CWT techniques^8,28,29^. We found oscillatory frequency intervals related to tissue/cell activity similar to those reported in human studies: myogenic (50–150 × 10^−3^ Hz), neurogenic (20–50 × 10^−3^ Hz), endothelial NO-dependent (9–20 × 10^−3^ Hz), endothelial NO-independent (5–9 × 10^−3^ Hz) (Fig. 3a). In contrast, cardiac (1350–5000 × 10^−3^ Hz) and respiratory (150–1350 × 10^−3^ Hz) components showed higher frequency ranges (Fig. 3a), confirming the faster heart and breathing rates expected for mice^30,31^. Although the magnitude of the oscillations could be reduced due to the effects of anaesthesia^32^, we used a light isoflurane anaesthesia to avoid major systemic effects on the circulation. Additionally, all mice were scanned under the same conditions thus making it possible to compare differences between groups.

CWT allows the identification of two oscillators reflecting ECs activity. While the biological link between the 9–20 × 10^−3^ Hz oscillator and the endothelial NO mechanism has been demonstrated^33^, the origin of the 5–9 × 10^−3^ Hz oscillator (endothelial NO-independent) is ambiguous. However, our results and literature reports^28,29^ suggest indirectly an association with the endothelial-derived hyperpolarizing factor (EDHF) vasodilation mechanism, thus here we will refer to the endothelial NO-independent component as EDHF. The reasons supporting this hypothesis are explained in the Supplementary Information.

### Characterisation of metabolic fluctuations

*In*-*vivo* metabolic oscillations were detected by CWT analysis of NAD(P)H_*normalised*_ and RR_*index*_ reconstructed signals. Figure 3b shows an example of CWT scalogram and the corresponding time-averaged spectrum from NAD(P)H signal. High spectral energy was found in the low-frequency ranges, thus slow oscillators dominate the reconstructed tracing. Although time resolution is scarce in the low frequencies, we clearly identified three heterogeneous oscillatory intervals defined metabolic oscillator-1 (MO-1) (5–9 × 10^−3^ Hz), metabolic oscillator-2 (MO-2) (2.5–5 × 10^−3^ Hz) and metabolic oscillator-3 (MO-3) (1.5–2.5 × 10^−3^ Hz). However, we will discuss only results of MO-1 because MO-2 and MO-3 frequency ranges fall largely at the edges of the cone of influence region in the CWT scalogram, where data might not be reliable^34^ (see caption of Fig. 3 for more details).

### LDF oscillators’ changes during vasomotion and comparison between groups

**Figure 4:**
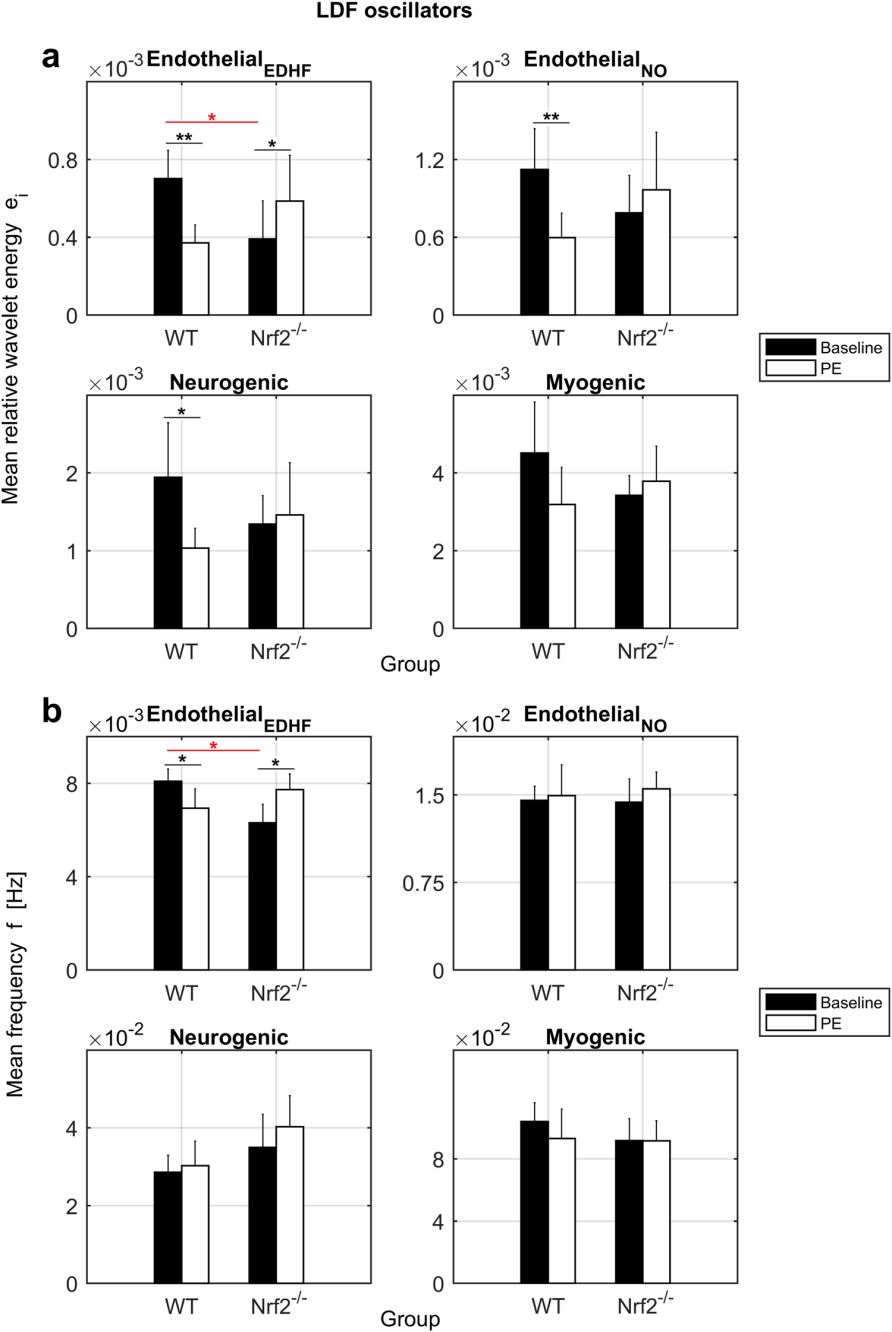
Results of LDF oscillators. (**a**) Mean relative energy *e*_*i*_ (dimensionless) of the wavelet peaks. (**b**) Mean frequency *f* (Hz) of the wavelet peaks. Error bars = 2 SE. Black bars = Baseline. White bars = PE. WT = Control. Nrf2^−/−^ = Knockout. Black lines/asterisks = Significant changes during PE administration. Red lines/asterisks = Significant differences between groups. t-test *p < 0.05, **p < 0.01.

Figure 4 describes the quantitative results of the relative spectral energy *e*_*i*_ and frequency *f* extracted from the time-averaged wavelet peak of each LDF oscillator. The relative *e*_*i*_, to allow comparisons between groups, was obtained by normalising the absolute energy extracted from the peak of each oscillator (*E*_*i*_, area under the curve) by the total absolute energy *E*_*tot*_ of the wavelet spectrum and the number of frequencies in the interval of the oscillator.

The spectral *e*_*i*_ and frequency of the EDHF endothelial oscillator were significantly greater in WT mice at baseline (energy p = 0.03, frequency p = 0.005). The EDHF energy and frequency changed significantly in both groups during PE stimulation following opposite trends. The values increased in Nrf2^−/−^ mice (energy p = 0.01, frequency p = 0.02) and decreased in controls (energy p = 0.008, frequency p = 0.04). The endothelial NO oscillator displayed the same trends observed for the EDHF oscillation. However, we found statistical significance only for the decrease of energy during phenylephrine delivery in WT mice (p = 0.008). The neurogenic and myogenic components showed patterns similar to those of the endothelial oscillations. The energy was reduced in normal mice after PE administration, with a good p-value for the neurogenic oscillator (p = 0.04) and a p-value close to statistical significance for the myogenic component (p = 0.08). We evaluated also the relative maximal amplitude *a*_*i*_ of the wavelet peaks, estimated by normalising the absolute amplitude *A*_*i*_ of each oscillator by the total amplitude *A*_*tot*_ of the wavelet spectrum and the number of frequencies in the interval of the oscillator. The data of *a*_*i*_ are summarised in Table 1 and displayed the same patterns found for *e*_*i*_ but with lower statistical significance.

These results outline opposite microvascular reactivity to PE in WT and Nrf2^−/−^ mice, especially for the endothelial components that are key factors determining microvascular function. This suggests that oxidative stress may affect endothelial function and the overall cardiovascular dynamics in the long term.

In general, the increase of LDF oscillators’ spectral energy reflects vasodilation dynamics^28,29,33^, thus the expected oscillatory pattern during mild vasoconstriction (1% PE) should be a decrease of *e*_*i*_, as observed in WT animals. In contrast, Nrf2^−/−^ mice displayed an increase of LDF oscillators’ *e*_*i*_ during phenylephrine administration. The reason for this behaviour might be the activation of an endothelium-mediated vasomotion resistance mechanism to attenuate vasoconstriction defined myoendothelial feedback^15^, consistent with the alternation of vasoconstriction and vasodilation induced respectively by simultaneous targeting of VSMCs with PE and EDHF/NO endothelial signalling. The cause for the activation of this mechanism may be a general major vasoconstriction in micro-vessels affected by oxidative stress. We cannot exclude that also WT mice may activate this mechanism in response to higher vasoconstriction stimuli (PE > 1%).

### Metabolic oscillations’ changes and comparison between groups

**Figure 5:**
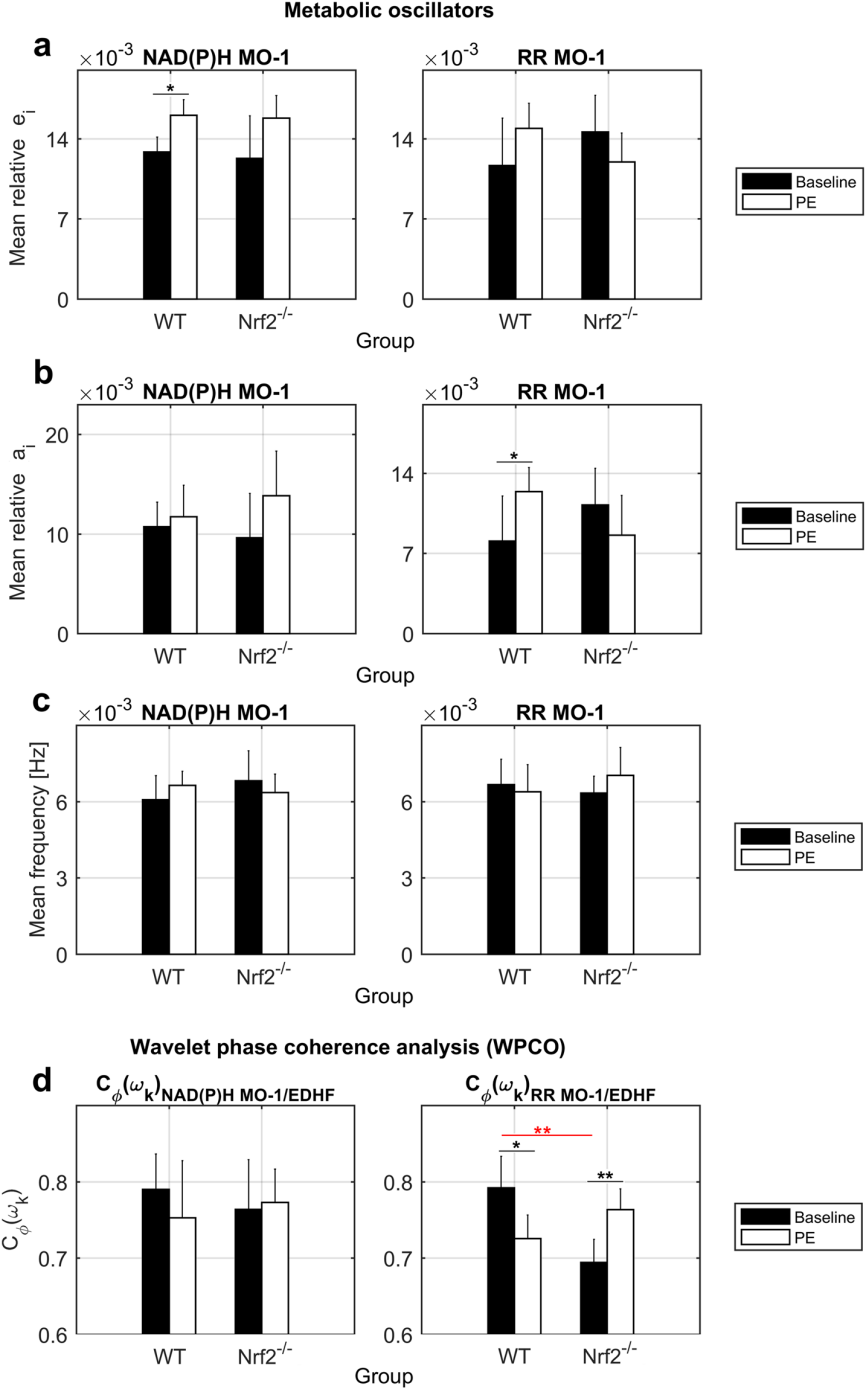
Results of metabolic oscillators and WPCO analysis. (**a**) Mean energy *e*_*i*_ (dimensionless), (**b**) mean amplitude *a*_*i*_ (dimensionless), and (**c**) mean *f* (Hz) of NAD(P)H and RR MO-1 wavelet peaks. (**d**) Average phase coherence C*ϕ*(*ω*k) (dimensionless) between NAD(P)H or RR MO-1 and the endothelial EDHF oscillator. The WPCO analysis is useful to study the phase relationship between low-frequency oscillators, which cannot be performed using the analysis of synchronisation due to the noisy background in the slow CWT components. Error bars = 2 SE. Black bars = Baseline. White bars = PE. WT = Control. Nrf2^−/−^ = Knockout. Black lines/asterisks = Significant changes during PE delivery. Red lines/asterisks = Significant differences between groups. t-test *p < 0.05, **p < 0.01.

Data of the spectral *e*_*i*_, *a*_*i*_ and *f* of NAD(P)H MO-1 and RR MO-1 wavelet peaks are summarised in Fig. 5a–c. Only WT mice showed a statistically significant increase of NAD(P)H MO-1 *e*_*i*_ (p = 0.01) and RR MO-1 *a*_*i*_ (p = 0.04) during PE delivery. The baseline RR MO-1 amplitude *a*_*i*_ was lower in WT compared to Nrf2^−/−^ mice (p = 0.097), while differences in frequencies were not relevant.

NAD(P)H_*normalised*_ and RR_*index*_ oscillations reflect the cellular oxido-reductive dynamics, consistent with the turnover between the oxidised and reduced forms of NAD(P)H and FADH_2_ coenzymes as part of the cyclic reactions for the production of ATP energy during glycolysis and oxidative phosphorylation (OXPHOS) processes. The results of the spectral analysis outline different dynamics of metabolic oscillators in WT and Nrf2^−/−^ mice, especially for the RR MO-1. Considering that RR_*index*_ reflects mitochondrial function, the major discriminatory power of RR MO-1 may indicate abnormal mitochondrial energetic dynamics in Nrf2^−/−^ mice due to increased amounts of reactive oxygen species (ROS) affecting the electron transport chain.

### Wavelet phase coherence (WPCO)

The WPCO analysis allows investigating the phase relationship C*ϕ*(*ω*k) between oscillations in a specific frequency range of two signals measured simultaneously. We used WPCO to explore the phase interaction between the endothelial EDHF oscillator and the MO-1 of NAD(P)H_*normalised*_ (C*ϕ*_*NAD*(*P*)*H* *MO*–1/*EDHF*_) or RR_*index*_ (C*ϕ*_*RR* *MO*–1/*EDHF*_) signals, which fall in the same low-frequency interval (5–9 × 10^−3^ Hz). Because our analysis does not include the surrogate data testing described recently by Gruszecki *et al*.^35^, we cannot make reliable assumptions on the degree of coherence at rest due to bias affecting the low frequencies. However, we can cautiously compare differences between mice phenotypes and changes in response to PE stimulation. Non-significant differences were found for the C*ϕ*_*NAD*(*P*)*H* *MO*–1/*EDHF*_ (Fig. 5d). In contrast, baseline C*ϕ*_*RR* *MO*–1/*EDHF*_ was significantly lower in Nrf2^−/−^ mice (p = 0.005), and changed significantly in both groups during PE administration showing a decrease in WT animals (p = 0.01) and an increase in Nrf2^−/−^ mice (p = 0.003) (Fig. 5d). These results outline again a major discriminatory power of RR MO-1 compared to NAD(P)H MO-1, suggesting that oxidative stress may impact the interaction/coupling between mitochondrial reactions and the EDHF vasodilation mechanism affecting vascular reactivity to PE.

### Relevant correlations

#### Correlations in WT mice

**Figure 6:**
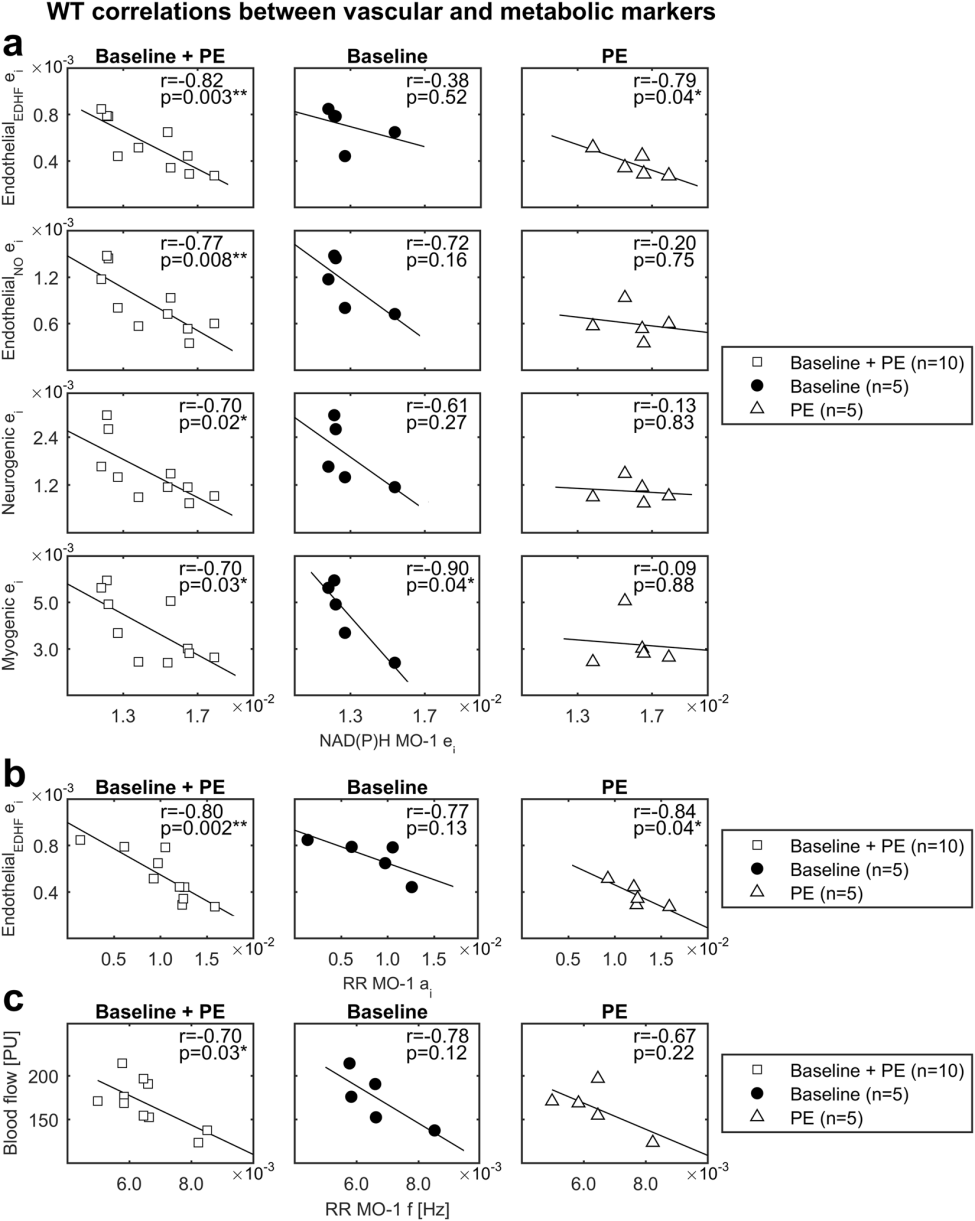
Correlations in WT mice. (**a**) Correlations between NAD(P)H MO-1 *e*_*i*_ and endothelial EDHF, endothelial NO, neurogenic, myogenic *e*_*i*_. (**b**) Correlation between RR MO-1 *a*_*i*_ and endothelial EDHF *e*_*i*_. (**c**) Correlation between RR MO-1 *f* and blood flow. *r* = Pearson’s correlation coefficient. White squares = Baseline + PE (pooled data). Black dots = Baseline. White triangles = PE. *p ≤ 0.05, **p < 0.01.

Figure 6 displays the correlations between microvascular and metabolic variables observed in WT mice. Due to the small number of animals (n = 5) available in the study, separating baseline and PE data in two distinct subgroups would reduce the statistical power, thus we have pooled all the data in a single correlation analysis. However, for completeness of information the results are presented for the pooled group (baseline + PE, n = 10) and the separate baseline and PE subgroups (n = 5, statistical power < 0.8). The pooled analysis revealed significant negative correlations between the EDHF *e*_*i*_ and both NAD(P)H MO-1 *e*_*i*_ (r = −0.82, p = 0.003) and RR MO-1 *a*_*i*_ (r = −0.80, p = 0.002). NAD(P)H MO-1 *e*_*i*_ showed also high negative correlations with the energy of endothelial NO (r = −0.77, p = 0.008), neurogenic (r = −0.70, p = 0.02) and myogenic (r = −0.70, p = 0.03) oscillators, and the RR MO-1 *a*_*i*_ was negatively related to blood perfusion (r = −0.70, p = 0.03). None of these correlations were observed in Nrf2^−/−^ mice. These results outline a robust association between the oxido-reductive fluctuations of NAD(P)H and FADH_2_ involved in ATP biosynthesis and the activation/inactivation of microvascular regulatory mechanisms. Indeed, the correlations probably indicate that in response to 1% PE the spectral energy of vascular oscillators decreases as the energy of slow metabolic oscillators increases. This may reflect a change in the turnover between NAD(P)^+^ and NAD(P)H promoting the inhibition of EDHF/NO endothelium-mediated vasodilation, and the enhancement of VSMCs-mediated vasoconstriction.

In spite of reduced statistical power, the analysis of correlations for the single baseline and PE subgroups provided useful additional information, indicating which are the correlations mostly associated with the baseline and PE time points. Significant negative correlations were found between the endothelial EDHF *e*_*i*_ and the NAD(P)H MO-1 *e*_*i*_ (r = −0.79, p = 0.04) or the RR MO-1 *a*_*i*_ (r = −0.84, p = 0.04) in the PE subgrop. These observations suggest that the metabolic oxido-reductive dynamics may be involved especially in the modulation of the EDHF pathway during *α*-adrenergic stimulation. In contrast, the endothelial NO and myogenic *e*_*i*_ showed a more significant correlation with NAD(P)H *e*_*i*_ in the baseline subgroup (r = −0.72, p = 0.16 and r = −0.90, p = 0.04 respectively).

#### Correlations in Nrf2^−/−^ mice

**Figure 7:**
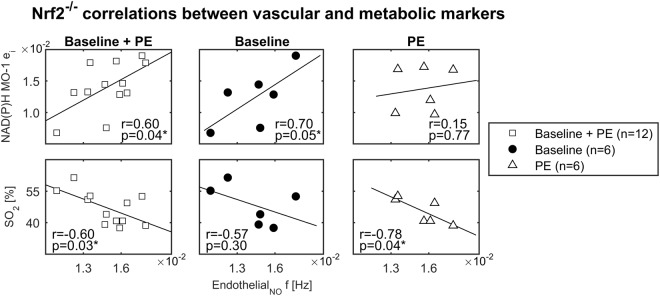
Correlations in Nrf2^−/−^ mice. Correlations between endothelial NO *f* and NAD(P)H MO-1 *e*_*i*_ or SO_2_. *r* = Pearson’s correlation coefficient. White squares = Baseline + PE (pooled data). Black dots = Baseline. White triangles = PE. *p ≤ 0.05, **p < 0.01.

Figure 7 displays the most important correlations observed in Nrf2^−/−^ mice. Also in this case due to the small number of animals (n = 6), the results are presented either as pooled group (baseline + PE, n = 12) and separate baseline and PE subgroups (n = 6). The main correlations found in the pooled group were related to the wavelet frequency *f* of the endothelial NO oscillator, which was positively correlated with NAD(P)H MO-1 *e*_*i*_ (r = 0.60, p = 0.04) and negatively correlated with SO_2_ (r = −0.60, p = 0.03). These correlations might reflect the overexpression of the endothelial nitric oxide synthase (eNOS) enzyme described previously in Nrf2^−/−^ mice^27^, leading to major production of NO probably to balance for dysfunction in other vasodilation pathways preserving vascular function in vessels affected by oxidative stress^27^. The correlations may be consistent with this evidence because both NADPH and molecular oxygen participate in the reaction for NO biosynthesis^36^. Therefore, the increase of endothelial NO frequency correlated with the decrease of SO_2_ and the increase of NAD(P)H MO-1 *e*_*i*_ might reflect respectively growth of NO production, consumption of molecular oxygen and oxidation of NADPH in NADP^+^. We could speculate that a sustained NO production may compensate for dysfunction in the EDHF vasodilator activity that was found significantly lower at baseline in mice affected by oxidative stress compared to WT (Fig. 4a). However, further research with a greater number of animals and a more appropriate model for the study of the NO pathway (e.g. eNOS knockout mice) is required to elucidate better these aspects. Indeed, the results of the baseline and PE subgroups showed a significant correlation between the endothelial NO frequency and NAD(P)H MO-1 *e*_*i*_ only at baseline (r = 0.70, p = 0.05), while the endothelial NO frequency and the oxygen saturation were significantly correlated only during PE delivery (r = −0.78, p = 0.04), which may indicate that the two correlations are not linked to the same physiological process.

## Discussion

In this work, cutaneous vasomotion and cell metabolic oscillations were examined simultaneously from live mice. A vasomotion phenomenon specifically mediated by NO and EDHF endothelial mechanisms^5^ was stimulated through administration of low-dose phenylephrine. Comparisons between Nrf2^−/−^ mice affected by oxidative stress and WT controls revealed an opposite behaviour of vascular oscillations in the two models, indicating an effect of oxidative stress on microvascular reactivity. The most relevant differences were found especially for the endothelial EDHF oscillator, and vasomotion was more prominent in Nrf2^−/−^ mice probably due to the activation of the myoendothelial feedback^15^ vascular modulation in response to oxidative stress.

We characterised a low-frequency metabolic oscillator (MO-1) of NAD(P)H_*normalised*_ and RR_*index*_ signals that displayed different dynamics in WT and Nrf2^−/−^ models, probably reflecting an alteration of the mitochondrial energetic processes due to oxidative stress. Relevant correlations were found in WT mice between metabolic and microvascular oscillators, highly suggesting an involvement of the cellular processes associated with fluctuations of NAD(P)H concentrations (i.e. OXPHOS and glycolysis) in microvascular reactivity. These correlations were absent in Nrf2^−/−^ mice suggesting an influence of oxidative stress on the interaction/coupling between cell metabolic reactions and microvascular regulatory mechanisms.

Our findings indicate indirectly that cell metabolic oscillators may have an important role in modulating vasomotion in response to specific stimuli at least for oscillatory processes endothelium-mediated. Therefore, this raises interest in the study of cell mitochondrial and glycolytic oscillators to elucidate further the mechanisms driving the Ca^2+^ oscillations at the basis of vasomotion. Despite ECs seemed the microcirculation component mostly associated with slow metabolic oscillators, metabolic fluctuations were also correlated with neurogenic and myogenic components indicating a global cooperation of multiple factors during vasomotion with higher/lower contributions depending on the type of stimulus and vascular bed. In the context of skin microcirculation, primary metabolic Ca^2+^ oscillations coming from ECs and the EDHF may represent the key drivers and regulatory factors for vasomotion. Based on our results, vasomotion can be considered an adaptive mechanism directed by the energetic requirements/stimuli in the environment surrounding blood vessels aimed at ensuring an optimal intake of nutrients (i.e. glucose) and oxygen for the production of ATP energy, rather than a process leading exclusively to the spontaneous pacemaker activity of VSMCs. This definition could help addressing better several controversial aspects, i.e. the observation of vasomotion in both healthy and diseased conditions^1,2^, or the primary involvement of ECs or VSMCs in different vascular beds^1,2^. These contrasting observations may be the result of variable energetic requirements and molecular signals in the parenchymal tissue surrounding the micro-vessels, depending on the degree of nutrition/oxygenation, the presence of a particular pathology, and the cell types involved. According to the literature, the factors released by microvascular ECs (NO, EDHF, etc.) can diffuse to the underlying parenchymal tissue and modulate the mitochondrial metabolism, the production of ROS and inflammation^37^. Thus, considering that the autofluorescence signals measured in this work have a prevalent epidermal origin, the correlations found between metabolic oscillations and vasomotion might also reflect the communication between skin cells and the microvascular network to fulfil the cutaneous metabolic needs in response to the *α*-adrenergic stimulus.

A shortcoming of the present research is the impossibility to extend the results on a general basis due to the small-scale design of the study and the restriction of the experiments exclusively to the WT and Nrf2^−/−^ models. As future perspective to increase the applicability on a broader basis, further studies are required on a larger number of animals and on additional mouse models. In particular, testing a larger number of Nrf2^−/−^ mice after challenging with high fat diet or after inducing diabetes would be useful to characterise the behaviour of the nonlinear biomarkers in the presence of an extensive oxidative stress load or a metabolic disease, and testing the biomarkers in a knockout model for the eNOS enzyme would be useful to elucidate better the role of NO.

The experiments in the present work were restricted to female mice due to limited availability of animals, thus another point to address in the future is also the extension of the study to male animals. The groups of tested mice were perfectly matched for an heterogeneous age range (36–60 weeks), which allowed making only genotype-related comparisons. However, this does not represent an issue because the main purpose of the current study was exploring the relationship between metabolic and microvascular oscillations in normal physiology and then use an example where metabolic function was disturbed (Nrf2^−/−^ model). As future perspective, a longitudinal study using more homogeneous aged animals could be also of interest to characterise the patterns of the nonlinear biomarkers for different age ranges.

The methodology used in this work can be easily translated for the concurrent study of metabolic and microvascular dynamics in humans and the exploration of metabolic oscillators as cardiovascular risk factors for diagnostic purposes. The use of LFS to monitor NAD(P)H provided some advantages, i.e. label-free minimally invasive nature of the measurements, convenient combination of LFS and LDF probes for simultaneous recordings, portability of the technology. However, there are limitations leading to the discrete nature of LFS measurements, necessity to reconstruct NAD(P)H_*normalised*_ and RR_*index*_ signals to characterise the oscillators, and the inability to trace the specific cellular origin of the oscillations. Furthermore, the technique was restricted to the investigation of low-frequency fluctuations due to discrete slow sampling of the spectra. More advanced technologies for the *in*-*vivo* imaging of NAD(P)H, i.e. fluorescence lifetime-based methods^38–40^ and multiphoton microscopy^25,41–43^, allow a better monitoring and discrimination between glycolytic and mitochondrial NAD(P)H with high spatial-temporal resolution at cell level. Combining these methods with CWT can help studying both slow and fast NAD(P)H oscillators. Nevertheless, these technologies still require further implementation for the application on humans and for providing continuous NAD(P)H recordings, they are expensive and lacking of portability and device design for combining with the LDF probe to study simultaneously metabolic and microvascular oscillators.

## Methods

### Animals

Animal work was conducted at the School of Medicine of the University of Dundee, after gaining approvals by the local ethics committee (University of Dundee Ethical Review Process). All the experimental procedures were performed in accordance with the UK Home Office relevant guidelines and regulations under the auspices of Project Licence PPL No. 60/4265. Animals included in the research were WT (n = 5) and Nrf2^−/−^ (n = 6) female mice on a C57Black/6 background aged 36–60 weeks. Mice were maintained under a 12:12-h light/dark cycle at 22.0 ± 1.0 °C and 50% humidity, and were fed ad libitum on a standard rodents chow diet regime and water.

### Skin preparation and anaesthesia

LFS and LDF techniques are sensitive to light absorption by hair and skin pigmentation. Therefore, a hair-free non-pigmented intact skin is required to obtain reliable measurements. Forty-eight hours prior to performing the experiments, hair from the mice flanks was shaved using an electric shaver, and the residual hair was removed using depilatory cream (Veet, Reckitt-Benckiser). Before collecting measurements, mice were anaesthetised through a standard Boyle’s Apparatus to prevent movement artefacts and were laid on a heat mat at 37.0 °C, as described previously by us^44^. A light anaesthesia was maintained by delivering 1.5–2% isoflurane (Abbott Laboratories) in oxygen (1.5 L/minute) through an inhalation nose cone (Fig. 1a).

### Iontophoresis

Iontophoresis allows the local delivery of vasoactive drugs in the skin microcirculation without inducing systemic effects. The drug is transferred transdermally by the unidirectional movement of ions in a solution, through a continuous current generated by a reference electrode and a platinum electrode incorporated in a ring-shaped chamber^44,45^. We used an iontophoresis chamber (ION6 probe, Moor Instruments, UK) of 20 mm internal diameter attached to the mouse flank using double-adhesive tape, and a reference electrode placed underside of the animal. The chamber was filled with a 2 ml 1% phenylephrine solution, and LFS and LDF probes were placed at two adjacent sites on the skin area inside the chamber (Fig. 1a). LFS/LDF signals were measured simultaneously for 20 min, 10 min without application of electric current (baseline), and 10 min during delivery of PE by applying a continuous 100 *μ*A anodal current through a controller (MIC2, Moor Instruments, UK) connected to the electrodes.

### Laser Fluorescence Spectroscopy

LFS employs a low-power laser (1–5 mW) for the *in*-*vivo* excitation of cell endogenous fluorophores, i.e. NAD(P)H, allowing the detection of autofluorescence emission peaks proportional to the tissue concentration of the biomarkers^46–48^. We assessed skin NAD(P)H autofluorescence with a single-point LFS probe (LAKK-M, Spe Lazma, Russia) provided with irradiating and detection sensors at a distance of ~1 mm for targeting a tissue volume of ~1 mm^3^. Discrete 10 seconds autofluorescence spectra were sampled from the mouse flank by a 365 nm UV excitation light (1 mW) at a rate of one spectrum every minute during iontophoresis. The spectra were processed by Matlab R2015a (The MathWorks Inc.) to extract the amplitudes of NAD(P)H (490 nm), FAD^+^ (550 nm), and elastin (450 nm) peaks (Fig. 1b). The redox ratio and normalised NAD(P)H autofluorescence were estimated respectively according to equations 1 and 2. For the study of NAD(P)H_*normalised*_ and RR_*index*_ oscillations by CWT, 20 min signals were reconstructed from the discrete data points (Fig. 1c) using the piecewise cubic spline interpolation method that we have previously described for reconstructing blood flow signals^49,50^. More details on signals reconstruction and NAD(P)H autofluorescence normalisation are provided in the Supplementary Information.

### Laser Doppler Flowmetry and oxygen saturation

LDF allows the indirect measurement of blood perfusion by sensing the Doppler-shift in wavelength generated when a monochromatic and coherent light is backscattered by moving blood cells^7,8,28,33,45^. We measured skin blood flow using a laser Doppler flowmeter (LAKK-M, Spe Lazma, Russia) provided with a probe identical to the LFS probe described above, which was calibrated with a fluoroplastic oscillating disk simulating Brownian motion. A 20 min LDF signal was measured during iontophoresis (Fig. 1d) using a 1064 nm laser with sampling frequency of 20 Hz. The multi-functional probe was also provided with 630 nm and 532 nm lasers, which allowed measuring the percentage skin oxygen saturation (SO_2_) according to the reflectance spectroscopy principles^51^. The technique is based on the analysis of the signal backscattered from the skin to detect the differential absorption of red and green visible lights by the oxygenated (oxyHb) and deoxygenated (deoxyHb) haemoglobin (Hb). The absorption is proportional to the relative molar concentrations of Hb fractions in the tissue, which are extracted from the backscattered spectrum by application of the Beer-Lambert law^52^:

3 $${\mu }_{a}(\lambda )=\sum _{i}\,{\varepsilon }_{i}(\lambda )\cdot {C}_{i},$$

where *ε*_*i*_(*λ*) is the molar extinction coefficient of each absorber, C_*i*_ is the molar concentration of each absorber, and *μ*_*a*_(*λ*) is the absorption coefficient. The percentage SO_2_ is finally estimated as follow^52^:

4 $$S{O}_{2}=\frac{[oxyHb]}{[oxyHb+deoxyHb]}\cdot 100 \% .$$

More details and references on how the terms of equations 3 and 4 can be extracted from the backscattered signal are provided in the paper by Dunaev *et al*.^52^.

### Continuous Wavelet Transform

The CWT method is largely employed to analyse blood perfusion vasomotion dynamics in the time-frequency domain^8,28,29^, providing optimal frequency resolution for low frequencies and good time resolution for high frequencies. We employed this method to investigate heterogeneous oscillations of LDF, NAD(P)H_*normalised*_ and RR_*index*_ signals, using a CWT with scaling factor *s*, time *t*, Morlet wavelet function *ψ* and central frequency 1^8,28^:

5 $$g(s,t)=\frac{1}{\sqrt{s}}\,{\int }_{-\infty }^{\infty }\,\psi (\frac{u-t}{s})g(u)du.$$

CWT and the extraction of the spectral energy *E*_*i*_^28^, amplitude *A*_*i*_^28^ and frequency *f*^28^ from the time-averaged wavelet peaks were performed using Matlab R2015a (The MathWorks Inc.). Figure 3 shows examples of CWT scalograms and corresponding time-averaged spectra from LDF and NAD(P)H_*normalised*_ signals.

### Wavelet phase coherence

The WPCO analysis provides information on the phase relationship C*ϕ*(*ω*k) between oscillators in the same frequency interval of two signals recorded simultaneously^53^. The analysis returns values between 0 and 1, where C*ϕ*(*ω*k) ≈ 0 indicates absence of coherence, C*ϕ*(*ω*k) ≈ 1 complete coherence, and 0 < C*ϕ*(*ω*k) < 1 partial coherence^53^. We investigated the coherence between MO-1 of metabolic signals and the LDF endothelial EDHF oscillator that are located in the same frequency range. The analysis was performed through the WPCO code provided in the Lancaster University website http://py-biomedical.lancaster.ac.uk by Professor Aneta Stefanovska research group, which estimates C*ϕ*(*ω*k) according to the principles described by Bandrivskyy *et al*.^53^ and Clemson *et al*.^54^.

### Statistics

Statistical analyses were performed by R-Studio software. The Shapiro-Wilk test revealed normal distributions for all the variables tested in the study. Therefore, the correlations were evaluated by calculating the Pearson’s correlation coefficient *r*, differences between baseline and PE time points were tested by paired t-test, and differences between groups were assessed by unpaired t-test. The correlations were considered relevant for p ≤ 0.05 and values of *r* > 0.5 or < −0.5. The t-test was considered significant at p < 0.05 with a statistical power of 0.8.

## Supplementary information

Supplementary information

## Data Availability

All data generated or analysed during this study are included in this published article and its Supplementary Information file.
