# Supplementary material for: In-vivo correlations between skin metabolic oscillations and vasomotion in wild-type mice and in a model of oxidative stress
Source: Sci Rep. 2019 Jan 17;9:186. doi: 10.1038/s41598-018-36970-4 (PMC6336806; doi:10.1038/s41598-018-36970-4)
Supplement: Supplementary file 1 — Supplementary information [file 41598_2018_36970_MOESM1_ESM.pdf]

Supplementary information

In-vivo correlations between skin metabolic oscillations and vasomotion in wild-type mice and in a model of oxidative stress

Salvatore Smirni[1,*], Alison D. McNeilly[1], Michael P. MacDonald[1,2], Rory J. McCrimmon[1] & Faisel Khan[1,*]

[1]School of Medicine, Ninewells Hospital, University of Dundee, Scotland, UK
[2]School of Science and Engineering, University of Dundee, Scotland, UK
*Corresponding authors: salvatore.smirni@gmail.com
f.khan@dundee.ac.uk

Biological origin of the LDF endothelial nitric oxide (NO)-independent wavelet oscillator

The physiological origin of the endothelial NO-independent wavelet oscillator (5-9.5 × 10^{-3} Hz) of skin LDF signals has not been completely elucidated. The endothelial origin of this oscillator has been proved by experiments showing relevant changes of its wavelet amplitude during the cutaneous administration of acetylcholine (ACh) [1, 2], a vasoactive agent able to stimulate multiple endothelium-mediated vasodilation mechanisms including NO, prostaglandins (PGs) and the endothelial-derived hyperpolarizing factor (EDHF) pathways. Moreover, blocking both NO or PGs mechanisms respectively by administration of L-NMMA (N^G-monomethyl-l-arginine) and aspirin did not affect the endothelial NO-independent oscillation [1, 2]. This evidence suggests indirectly a more likely biological link of this oscillator with an endothelial vasodilation mechanism different from NO and PGs that could be with high probability the EDHF pathway.

The results that we have presented in this paper might support indirectly this hypothesis. Indeed, according to Okazaki *et al.* [3], the vasoconstriction stimulated by administration of phenylephrine (PE) in rat mesenteric artery is able to enhance a vasomotion phenomenon mediated specifically by NO and EDHF mechanisms but not by PGs. Even if in this work we have tested micro-vessels different from the rat mesenteric artery (skin micro-vessels), the results seem to agree with what has been reported by Okazaki *et al.* [3]. Indeed, as we have discussed in the Figure 4 of this paper, the oscillators showing the most statistically significant changes during PE stimulation were the endothelial NO and endothelial NO-independent components. Considering that the NO origin of the 9-20 × 10^{-3} Hz oscillation has been already recognised [4], by exclusion, the high significant changes that we found in the 5-9 × 10^{-3} Hz interval after PE stimulation should be ascribed to the EDHF activity. Furthermore, according to the literature, EDHF plays a more important role than NO in the reactivity of small resistance arterioles [5, 6, 7, 8]. Our results, discussed in the Figure 4 of the manuscript, have displayed changes that are more significant for the endothelial NO-independent frequency range compared to the endothelial NO interval, which would support an EDHF origin of this oscillator and its major involvement in the reactivity of small arterioles [5, 6, 7, 8].

Additional indirect proof of the possible EDHF origin for the endothelial NO-independent oscillator was also provided by experimental data that we have obtained from human subjects. We have measured blood flow signals by Laser Doppler Flowmetry (LDF) from the forearm of 40 individuals during a 25 min post-occlusive reactive hyperaemia (PORH) functional test, using the same LDF single-point probe employed in this study (LAKK-M, Spe Lazma, Russia) that we have already described in the methods section. All of the tested subjects signed an informed consent form before performing the experiment, as part of a research project approved by the University of Dundee Research Ethics Committee (UREC, Study n. 15064) according to the guidelines of the Declaration of Helsinki. The PORH test was divided in three main steps (supplementary **Figure S1**):

(1) Measurement of the LDF signal for 10 min at rest (baseline).
(2) Blocking blood flow through the brachial artery for 5 min, by inflating a pressure cuff placed in the upper part of the tested arm at 200 mmHg. This step is characterised by a massive decrease of the LDF signal because of a temporary ischaemia condition induced by the occlusion of flow.
(3) Release of the cuff's pressure and monitoring for 10 min the PORH response stimulated by the previous occlusion of flow. PORH response is consistent with a massive increase of blood flow up to a peak for the fast reperfusion of the cutaneous tissue after a temporary period of occlusion, and the subsequent restoration of the basal blood flow.

All the LDF signals collected during PORH stimulation from the 40 tested subjects were processed by continuous wavelet transform (CWT) spectral analysis to determine the contribution of each LDF oscillator to skin PORH response. The CWT analysis was performed according to the same methodology that we have already discussed in the methods section of

this paper. The results have revealed a median percentage increase of wavelet amplitude/energy during PORH compared to baseline much higher for the endothelial NO-independent (EDHF) oscillator (58.3-72.3%) compared to the other LDF oscillators (supplementary **Table S1**). According to the literature, the EDHF mechanism is the major mediator contributing to human skin PORH response [9], thus considering that the endothelial NO-independent wavelet peak showed the highest contribution to the cutaneous hyperaemic response our findings represent a robust indirect proof of the EDHF origin of this oscillator. As future perspective, direct proof may be provided from either mouse models or humans by monitoring the behaviour of this LDF wavelet oscillator during the inhibition of the EDHF vasodilation mechanism. This task can be achieved in mouse models by blocking EDHF through a combination of the inhibitors of K^+ channels apamin (APA) and charybdotoxin (ChTX) [10]. Examples of pharmacological agents that might be applied for the same purpose in human skin are the fluconazole or sulfaphenazole for blocking locally the EDHF mechanism mediated by the epoxyeicosatrienoic metabolites (EETs) [11], and the tetraethylammonium (TEA) for the local inhibition of the EDHF mechanism mediated by KCa channels [12].

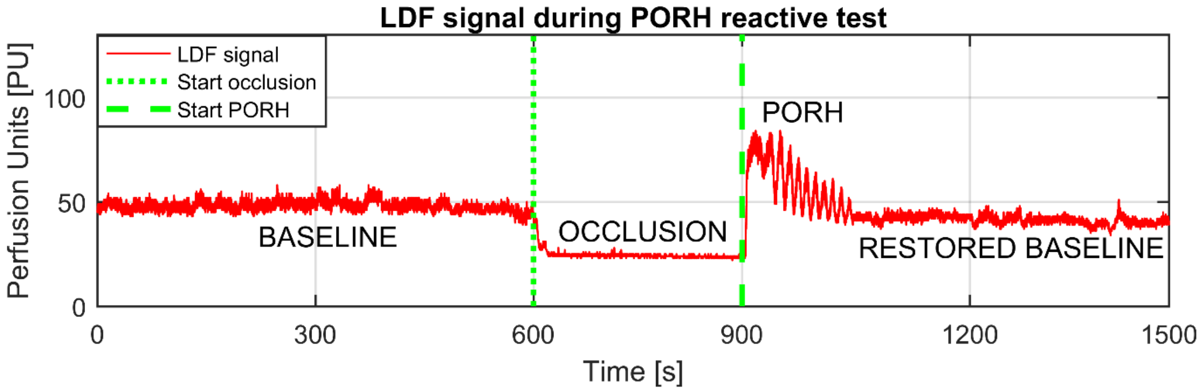

Figure S1. Example of LDF signal collected from the human forearm during PORH test. Data are expressed in perfusion arbitrary units (PU). The graph shows the typical trend of LDF signal during a 25 min PORH test. The first 10 min represent the baseline blood flow. Then the pressure cuff was inflated at 200 mmHg to induce an ischaemia period of 5 min characterised by decrease of flow. Finally, the pressure of the cuff was released to monitor for 10 min PORH response and the restoration of the resting blood flow.

Table S1. Percentage increase of the relative wavelet amplitude a_i and energy e_i of LDF oscillators during skin PORH response. The Shapiro-Wilk test performed by R-Studio software has revealed non-parametric distributions for all the variables. Thus, data are reported as median (inter-quartile range). n = Number of tested subjects. The increase of wavelet amplitude/energy was evaluated as percentage growth during PORH response compared to baseline.

PARAMETERS	n=40
Variable name	Median (inter-quartile range)
e_i Endothelial EDHF increase [%]	58.3 (48.3-79.3)
e_i Endothelial NO increase [%]	27.2 (14.0-33.1)
e_i Neurogenic increase [%]	5.60 (1.70-11.2)
e_i Myogenic increase [%]	5.50 (0.90-7.00)
a_i Endothelial EDHF increase [%]	72.3 (57.7-89.8)
a_i Endothelial NO increase [%]	18.3 (8.10-28.0)
a_i Neurogenic increase [%]	1.30 (0.10-7.50)
a_i Myogenic increase [%]	2.40 (0.50-6.00)

Reconstruction of NAD(P)H and RR time series by piecewise cubic spline interpolation

In this work, the *in-vivo* investigation of metabolic oscillations from mouse skin was achieved by the application of the CWT spectral analysis on $NAD(P)H_{normalised}$ and RR_{index} signals. While the LDF method is characterised by a fast sampling rate of perfusion values that provides blood flow tracings suitable to study microvascular oscillators using the CWT analysis, the LFS measures only single discrete autofluorescence (AF) spectra. For this reason, to allow characterising also the metabolic oscillations by using the CWT, NAD(P)H and RR time series were reconstructed from the discrete UV spectra collected over time ensuring to use the same number of samples as the simultaneous LDF tracing (one sample every 0.05 s). The reconstruction was performed in two steps by using the software Matlab R2015a (The MathWorks Inc.), according to the same methodology previously described by us for the reconstruction of blood flow signals from discrete data points [13]. First, the values of $NAD(P)H_{normalised}$ (equation 2 in the manuscript) and RR_{index} (equation 1 in the manuscript) were extracted from the 20 sequential UV AF spectra measured over time during iontophoresis test. Finally, the values were used as discrete data points to reconstruct a curve covering the whole 20 min period used in the experiment for acquiring the spectra. The reconstruction was performed by piecewise cubic spline interpolation,

applying an interval of 1 min between each pair of data points that corresponds to the sampling frequency adopted during the temporal collection of the AF spectra. The curve was reconstructed with a number of samples (n=24000) equal to those of the simultaneous LDF tracing (one sample every 0.05 s) to allow performing correctly the phase coherence analysis and comparisons between microvascular and metabolic data related to a specific temporal window.

The spline interpolation technique is a mathematical procedure based on the use of a piecewise low-degree polynomial called spline for the approximation of a continuous function *g* by interpolating equally spaced discrete data points of the function [13]. The main advantage of this technique is the accurate approximation of a function over large intervals, avoiding and reducing errors during the interpolation process, i.e. the introduction of oscillatory artefacts between each pair of data points (Runge's phenomenon) which is typical of interpolation processes performed by using a single high-degree polynomial [13]. The spline interpolation process consists in subdividing the interval [a, b] where *g* is continuous in smaller sub-intervals, and approximating *g* in each sub-interval using low-degree polynomial pieces that define a composite spline function *s* [13]. In this work, we used a cubic (third-degree) spline function which is suitable for reconstructing continuous natural processes, and requires four terms to define a piecewise polynomial between each pair of data points, as displayed by the supplementary **equation S1** [13],

$$p_3(x) = ax^3 + bx^2 + cx + d, \qquad \text{(S1)}$$

where p_3 is the piecewise third-degree polynomial, and *a*, *b*, *c* and *d* are the four terms defining p_3. The final curve resulting from the cubic spline interpolation process is made of a number of pieces reconstructed between each pair of data points equal to *1-n*, where *n* is the number of data points [13]. Therefore, considering that the number of NAD(P)H$_{\text{normalised}}$ and RR$_{\text{index}}$ discrete values used in this study during the interpolation process was 20, we have obtained NAD(P)H$_{\text{normalised}}$ and RR$_{\text{index}}$ reconstructed curves made of 19 pieces (supplementary **Figure S2**).

Considering that the cubic spline interpolation method does not introduce oscillatory artefacts in the reconstructed signal [13], by using this technique we have ensured that the results of the subsequent CWT analysis were reflecting the real oscillatory behaviour of NAD(P)H$_{\text{normalised}}$ and RR$_{\text{index}}$ detected by autofluorescence recordings. Nonetheless, a limit of this technique is poor accuracy in the reconstruction of the right and left extremities of the signal [13]. Thus, to avoid artefacts at the edges of the signal the time series was cut at the extremities making sure that the starting and ending points of the curve were corresponding respectively to the first and last experimental discrete data points [13].

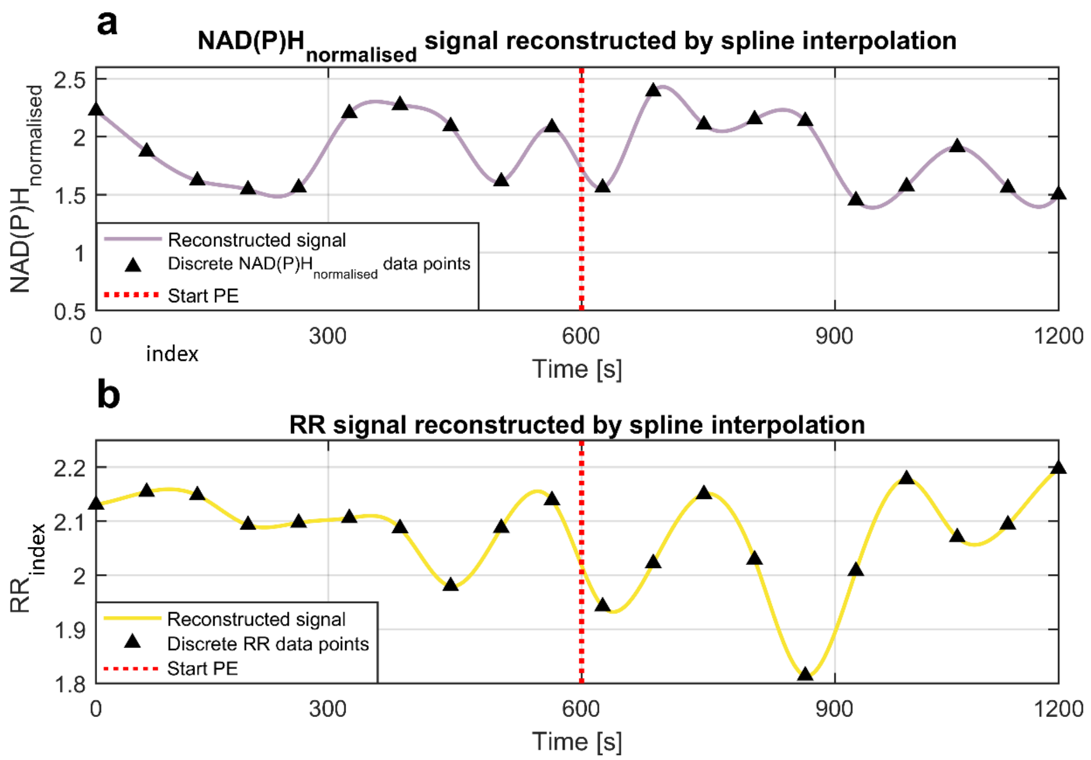

Figure S2. Example of signals reconstructed by cubic spline interpolation of discrete data points. (**a**) 20 min signal reconstructed using NAD(P)H$_{\text{normalised}}$ values extracted from 20 sequential discrete UV autofluorescence spectra measured from the flank of a mouse during a 20 min iontophoresis test. (**b**) 20 min signal obtained using RR$_{\text{index}}$ values estimated from 20 sequential discrete UV autofluorescence spectra measured from the flank of a mouse during a 20 min iontophoresis test. An interval of 1 min was applied between each pair of data points, which corresponds to the sampling frequency used for the temporal acquisition of the UV spectra (1 spectrum per minute). Both the reconstructed signals are made of a total of 24000 samples, and 19 pieces (*n-1*, where *n* is the number of discrete data points). The starting and ending points of the curve correspond respectively to the first and last experimental data points used for the reconstruction to avoid the presence of oscillatory artefacts at the extremities of the time series.

Normalisation of NAD(P)H to reduce blood volume and skin pigmentation effects

As already explained in the methods section of this paper, we have evaluated the relative changes of NAD(P)H concentrations and mitochondrial function (RR_{index}) from live mice skin by ratiometric normalisation of NAD(P)H fluorescence, respectively by the autofluorescence of the structural protein elastin (450 nm) and of the coenzyme FAD^+ (550 nm). The reason for the normalisation was reducing the effect of blood volume and cutaneous melanin on NAD(P)H autofluorescence signal for obtaining a more accurate assessment of cellular metabolic oscillations over time. Indeed, a major problem when evaluating the UV autofluorescence from *in-vivo* skin is that the signal is highly affected by artefacts due to absorption of UV light by cutaneous chromophores, i.e. haemoglobin (Hb) in the micro-vessel blood and melanin pigmentation [14, 15]. This is clearly visible in supplementary **Figure S3a-b** and supplementary **Figure S4a-b**, which show respectively the effects of blood volume changes and melanin on the reconstructed NAD(P)H autofluorescence signal obtained from measurements collected using the LAKK-M probe (Spe Lazma Ltd, Russia).

Supplementary **Figure S3** displays the trends of simultaneous median LDF (**Figure S3a**) and reconstructed NAD(P)H autofluorescence (**Figure S3b**) signals that we have measured from the forearm of human subjects (n=16) during a 25 min PORH test. The graphs clearly show a massive decrease of NAD(P)H autofluorescence signal intensity concurrent to the increase of blood flow/volume during PORH response. This observation clearly outlines the negative effect of blood volume changes on NAD(P)H signal due to absorption of UV light by Hb, which makes the measurements unreliable for the evaluation of NAD(P)H oscillations over time.

Supplementary **Figure S4** displays the trends of simultaneous median LDF (**Figure S4a**) and reconstructed NAD(P)H autofluorescence (**Figure S4b**) signals that we have measured from the forearm of subjects with dark skin (n=8) and white skin (n=8) during a 25 min PORH test. The graphs clearly show that while the colour of skin does not affect LDF measurements, the intensity of NAD(P)H autofluorescence signal is highly reduced in individuals with dark skin that is characterised by a major content of melanin pigmentation. However, while melanin absorption may represent a major problem for the evaluation of the absolute quantitative concentrations of NAD(P)H, also in this case the effect of blood volume seems to be the major obstacle for making a reliable assessment of the relative oscillatory changes of NAD(P)H over time that was the main goal of this work.

A widespread method used for the correction of NAD(P)H UV autofluorescence artefacts is the 1:1 ratio with another variable affected by the same artefacts, e.g. skin reflectance signal [14], in order to compensate for NAD(P)H unrelated factors obtaining a normalised signal which should reflect more reliably the relative temporal trends of NAD(P)H. In this work, we did not have the chance to measure skin reflectance signal, thus we have taken advantage of the overlapping contributions in the UV spectrum of elastin and FAD^+ autofluorescence to perform 1:1 ratiometric corrections of NAD(P)H autofluorescence.

Elastin is an abundant structural component in the connective tissue of the dermal cutaneous layer [15, 16]. The reason for using the autofluorescence of this protein as correction factor is that being a structural component it should maintain a constant amount in the cutaneous tissue during the "short" temporal period employed for the collection of autofluorescence data. Therefore, normalising the autofluorescence by elastin represents an intra-subject compensation for micro-environmental factors unrelated to NAD(P)H, i.e. Hb or melanin absorption, and the resulting signal should be more representative of NAD(P)H temporal dynamics due to the slow biological turnover of elastin and its constant amounts in the tissue during the temporal acquisition of the UV spectra. The autofluorescence emission of elastin after excitation with UV light is between 400-450 nm and could be affected by an overlapping contribution of collagen autofluorescence (390-460 nm) [15, 16]. However, considering that also collagen is a structural component abundant in the dermal connective tissue this should not represent a major problem because also this protein has a slow turnover and should present constant amounts in the skin during the temporal acquisition of the UV spectra.

Figure S3c shows the trend of NAD(P)H signal during PORH test after normalisation by elastin autofluorescence. The graph clearly displays a reduction of blood volume effect compared to **Figure S3b**, and a temporal trend of NAD(P)H that seems to reflect more reliably the relative changes in the concentrations of this coenzyme during PORH test. Indeed, $NAD(P)H_{normalised}$ signal increased during the 5 min occlusion of blood flow, probably reflecting the uncoupling of the electron transport chain and the inhibition of ATP production through the oxidative phosphorylation (OXPHOS) mitochondrial process due to reduced intake of oxygen and accumulation of reduced NAD(P)H [14]. In contrast, the moderate decrease of NAD(P)H during PORH response in this case may reflect the oxidation of NAD(P)H in $NAD(P)^+$ due to the restoration of a normal aerobic metabolism and increased production of ATP [14] rather than an artefact due to Hb absorption. Similar results are shown in **Figure S4c** demonstrating that $NAD(P)H_{normalised}$ signal is not affected by skin pigmentation and seems more reliable to compare the temporal oscillatory behaviour of NAD(P)H between cutaneous samples with different amounts of melanin.

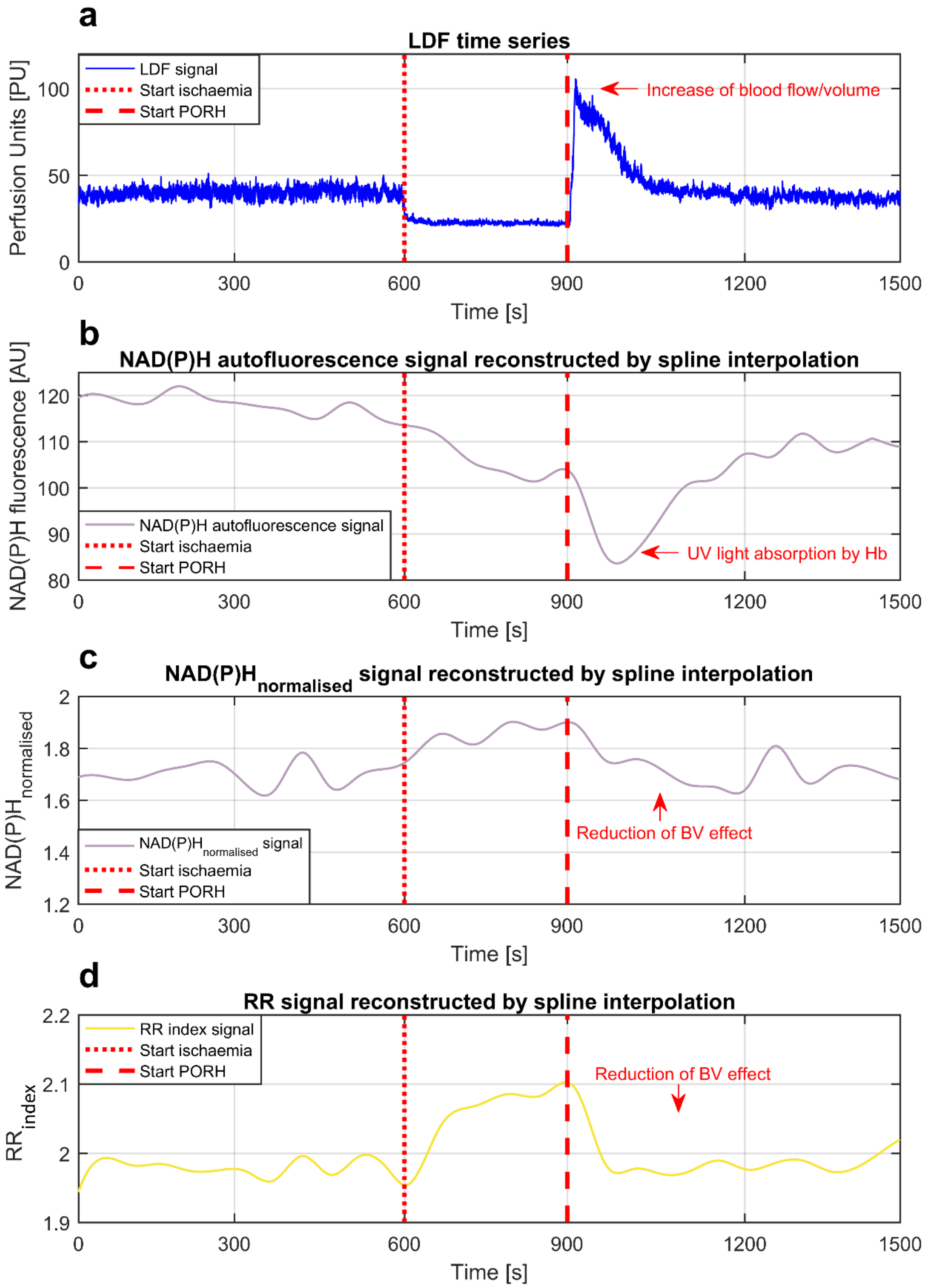

Figure S3. Correction of NAD(P)H autofluorescence signal to reduce blood volume effects. Examples of simultaneous median (**a**) LDF (PU) and (**b**) reconstructed NAD(P)H autofluorescence (AU) signals measured from the forearm of 16 human subjects during a 25 min PORH test. The graphs clearly display the absorption of UV light by Hb affecting NAD(P)H autofluorescence signal during PORH response due to increase of blood perfusion/volume. (**c**) 1:1 ratiometric normalisation of NAD(P)H signal by the autofluorescence of the structural protein elastin ($NAD(P)H_{normalised}$ expressed in dimensionless units). (**d**) 1:1 ratiometric normalisation of NAD(P)H signal by the autofluorescence of FAD^+ coenzyme (RR_{index} expressed in dimensionless units). Normalised signals reflect more reliably the trends of NAD(P)H and mitochondrial oxido-reductive dynamics expected during PORH functional test.

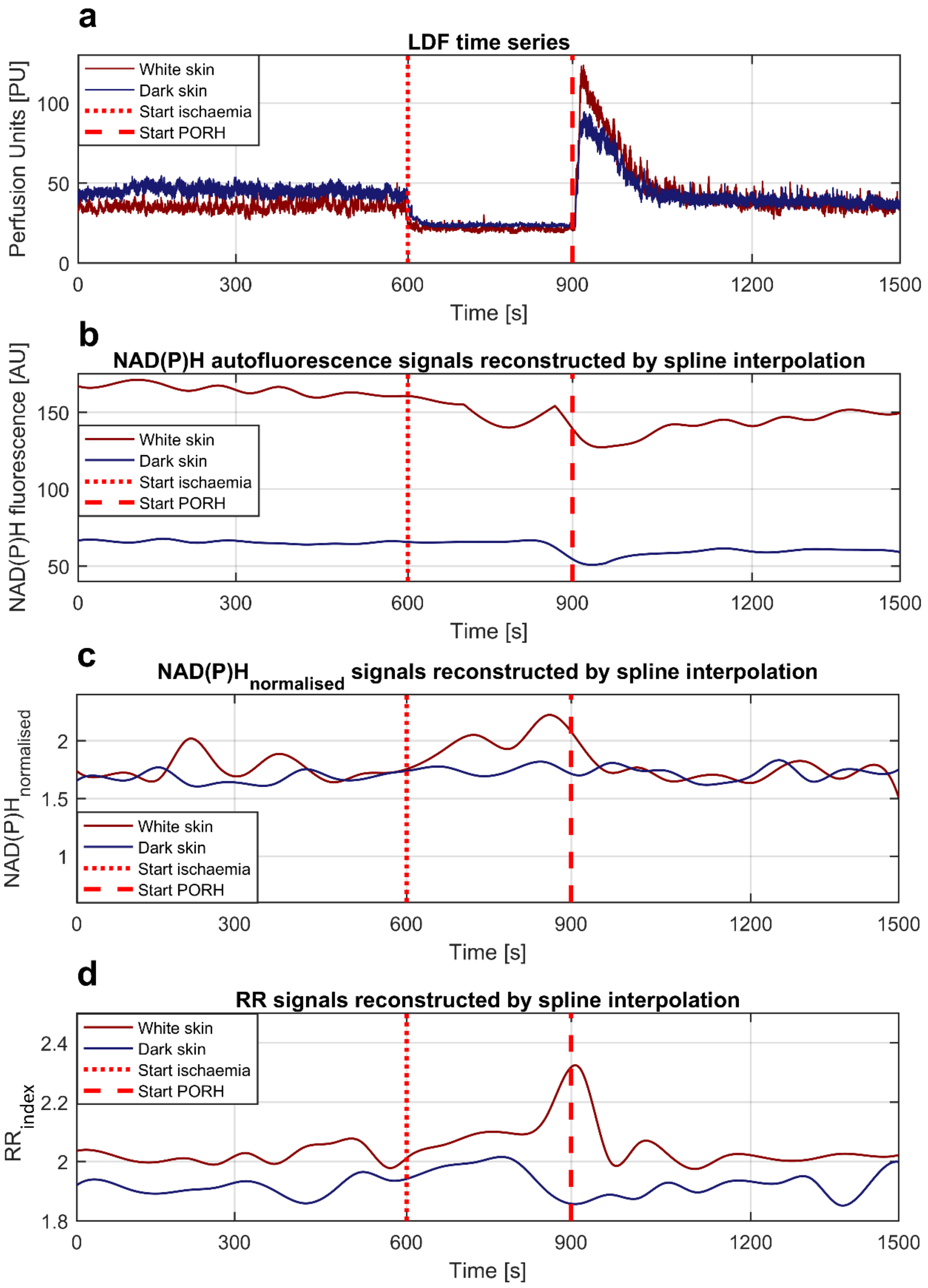

Figure S4. Effects of melanin pigmentation on NAD(P)H autofluorescence signal. Examples of simultaneous median (**a**) LDF (PU) and (**b**) reconstructed NAD(P)H autofluorescence (AU) signals measured from the forearm of 8 subjects with white skin (dark red lines) and 8 individuals with dark skin (dark blue lines) during a 25 min PORH test. The graphs clearly display that the LDF signal is not affected by different amounts of cutaneous melanin pigmentation. In contrast, the intensity of NAD(P)H autofluorescence signal was highly reduced in subjects with dark skin due to the major content of melanin absorbing UV light. (**c**) 1:1 ratiometric normalisation of NAD(P)H signals by the autofluorescence of the structural protein elastin ($NAD(P)H_{normalised}$ expressed in dimensionless units). (**d**) 1:1 ratiometric normalisation of NAD(P)H signal by the autofluorescence of FAD^{+} coenzyme (RR_{index} expressed in dimensionless units). Normalised signals were characterised by reduced blood volume effect reflecting more reliably the trends of NAD(P)H and mitochondrial oxido-reductive dynamics expected during PORH functional test, and seemed to be suitable for making acceptable comparisons of temporal oscillatory dynamics between subjects with different skin pigmentation.

The estimation of the redox ratio (RR_{index}) by normalising NAD(P)H autofluorescence for the fluorescence of the coenzyme FAD^+ provided also optimal results. This index is a measure of the balance between the reduced form of NAD(P)H and the oxidised FAD^+, which reflects specifically the activity of the mitochondrial electron transport chain and the degree of ATP energy production through the OXPHOS process [14, 17]. The interpretation of RR_{index} values may vary depending on the kind of stimulus affecting mitochondrial function, the degree of oxygenation, or the presence of particular pathologic conditions (e.g. cancer).

Figure S3d displays the trend of RR_{index} during PORH functional test, which seems to reflect reliably the changes of mitochondrial activity associated with a temporary ischaemia period and PORH response. The relevant increase of RR_{index} during ischaemia may reflect a preferential production of ATP through the glycolysis process in the presence of lower tissue oxygenation, due to accumulation of mitochondrial NAD(P)H and uncoupling of the electron transport chain. In contrast, the restoration of the baseline RR_{index} trend observed during PORH response may reflect the reactivation of the aerobic metabolism, oxidation of NAD(P)H in $NAD(P)^+$ and increase of ATP production through the OXPHOS cycle**.** **Figure S4d** displays the comparison of RR_{index} trends between subjects with dark coloured skin and individuals with white skin. The signals showed a trend similar to the RR_{index} signal in **Figure S3d**, reflecting the oxido-reductive mitochondrial dynamics expected during PORH test. Although the values of RR_{index} were slightly higher in the group of individuals with white skin, the signals seem to be comparable for an acceptable evaluation of the temporal RR_{index} oscillatory dynamics that was the main goal of this work.

The concurrent multi-parametric monitoring of LDF and autofluorescence signals during PORH test was helpful to optimise the correction of autofluorescence data, allowing the easy detection of non-physiological responses affecting NAD(P)H autofluorescence (i.e. effect of UV light absorption by Hb) and a better explanation of the physiological processes reflected by NAD(P)H signal. The application of the ratiometric normalisation approach provided time series with an acceptable degree of reliability for the evaluation of temporal metabolic oscillations, thus we decided to apply the same correction method on mouse models data presented in this work. Nonetheless, although the correction techniques used in this study allowed performing an acceptable evaluation of NAD(P)H oscillations, the development of more accurate, successful, and practical correction methods is still challenging. A promising approach for the future that we are pursuing is the Monte Carlo modelling of skin autofluorescence measurements to determine the interaction of the UV light with the cutaneous tissue, and the path of the photons detected by the single-point probe used for the measurements. This approach may help to characterise and quantify better the effects of blood volume and melanin on the autofluorescence signal and the implementation of more precise correction methods.

Spatial resolution of LFS measurements and cellular origin of the metabolic oscillators

A shortcoming of laser fluorescence spectroscopy (LFS) is the inability to quantify the contribution of specific groups of cells to the detected autofluorescence signal, due to the uncertain spatial resolution of the measurements and the heterogeneous structure of skin. Indeed, the cutaneous tissue includes cells from different layers (e.g. epidermal cells) and functional components such as micro-vessels (i.e. endothelial cells and vascular smooth muscle cells). Therefore, this makes it difficult also the characterisation of the cellular origin of the metabolic oscillators investigated in this work.

According to the literature, The 365 nm UVA wavelength has a penetration depth incorporating the human upper dermal skin layer [18, 19], where capillaries and arterioles are located. In mice, the epidermis is thinner than in humans [20], thus the UVA light should target the core of the dermal layer. This suggests that a relevant part of the detected autofluorescence signal might derive from the microcirculation network, which was the main tissue of interest in this research. The relevant correlations that we have found in this work between microvascular and metabolic oscillations may represent an indirect proof that part of skin metabolic oscillations is of microvascular origin. However, the correlations could mostly reflect the activation of vasomotion in response to the energetic/nutritive requirements and oscillatory metabolic stimuli coming from cutaneous cells (e.g. epidermal cells). The hypotheses above are supported by ongoing studies that we are performing in collaboration with a research group of the University of St. Andrews (Scotland, UK), based on Monte Carlo modelling of the penetration depth of the 365 nm UV laser that we have used in this study (LAKK-M, Spe Lazma Ltd, Russia). The simulation was performed in a 5-layer human skin model including various absorbers and scatterers in each stratum (supplementary Table S2), and taking into account the specific geometric parameters and wavelength of the LAKK-M UV probe: 365 nm source wavelength, 0.4 mm beam radius, 0.4 mm detector radius, and 1 mm probe-detector center separation. The simulation was performed considering a 75% tissue blood oxygenation, and using a model of Fitzpatrick skin type I aged ~30 years with a thickness similar to the skin at 1/3 length of the human forearm. The results of the simulation revealed a penetration depth of the UV laser around 200 µm (supplementary Figure S5), even though the graph suggests that ~99% of the photons are coming from a depth of 100 µm. In general, these findings support both an epidermal and a dermal origin of skin autofluorescence stimulated

by the LAKK-M UV laser. However, according to the simulation most of the autofluorescence signal measured in the human forearm is of epidermal origin (100 µm) with a partial smaller contribution of the papillary dermis region (200 µm) where the capillaries are located. Considering that mouse cutaneous tissue has a thinner epidermis than human skin, it can be hypothesised a greater contribution of the upper dermis and of the microvascular network to the autofluorescence signal in mouse models compared to humans.

Table S2 Human skin model used to simulate the fluence of the LAKK-M UV light. The model was structured in five layers: stratum corneum, epidermis, papillary dermis, reticular dermis and hypodermis. Each stratum was populated with various tissue absorbers and scatterers at specific concentrations: water, blood, melanin, bilirubin, β-carotene, and a baseline absorber. Data were kindly provided by Mr Lewis McMillan and Dr Kenneth Wood from the Astronomy group at the University of St. Andrews (Scotland).

SKIN LAYER	THICKNESS	ABSORBERS
Stratum corneum	0.030 mm	5% water, baseline absorber
Epidermis	0.080 mm	20% water, 0.5% melanin, 2.1 × 10^{-4} g/L β-Carotene, baseline absorber
Papillary Dermis	0.180 mm	50% water, 0.05 g/L bilirubin, 7 × 10^{-5} g/L β-Carotene, 6% blood, baseline absorber
Reticular Dermis	1.800 mm	70% water, 0.05 g/L bilirubin, 7 × 10^{-5} g/L β-Carotene, 4.5% blood, baseline absorber
Hypodermis	2.900 mm	70% water, 7% blood, baseline absorber

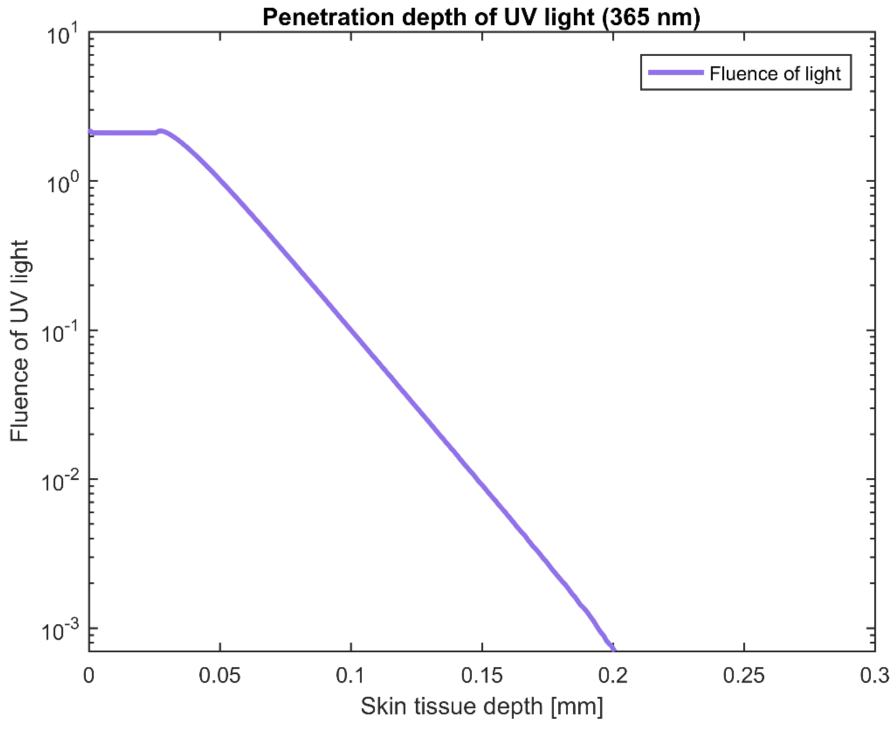

Figure S5. Monte Carlo modelling of the LAKK-M UV laser light propagation. A total penetration depth of ~200 µm (human upper dermis) was predicted with most of the light (~99%) coming from a depth of 100 µm (human epidermis). Data were kindly provided by Mr Lewis McMillan and Dr Kenneth Wood from the Astronomy group at the University of St. Andrews (Scotland, UK).

References

[1] Shiogai, Y., Stefanovska, A. & McClintok, P. V. Nonlinear dynamics of cardiovascular ageing. *Phys. Rep.* **488(2-3),** 51-110 (2010).

[2] Kvandal, P. *et al.* Low frequency oscillations of the laser Doppler perfusion signal in human skin. *Microvasc. Res.* **72(3),** 120-127 (2006).

[3] Okazaki, K. *et al.* Role of the endothelium-derived hyperpolarizing factor in phenylephrine-induced oscillatory vasomotion in rat small mesenteric artery. *Anesthesiology*. **98(5),** 1164-1171 (2003).

[4] Stefanovska, A., Bračič, M. & Kvernmo, H. D. Wavelet analysis of oscillations in the peripheral blood circulation measured by laser Doppler technique. *IEEE Trans. Biomed. Eng.* **46(10),** 1230–1239 (1999).

[5] Kapela, A., Nagaraja, S., Parikh, J. & Tsoukias, M. Modeling Ca2+ signaling in the microcirculation: intercellular communication and vasoreactivity. *Crit. Rev. Biomed. Eng.* **39(5),** 435-460 (2011).

[6] Shimokawa, H. *et al.* The importance of the hyperpolarizing mechanism increases as the vessel size decreases in endothelium-dependent relaxations in rat mesenteric circulation. *J. Cardiovasc. Pharmacol.* **28(5),** 703-11 (1996).

[7] Hwa, J. J., Ghibaudi, L., Williams, P. & Chatterjee, M. Comparison of acetylcholine-dependent relaxation in large and small arteries of rat mesenteric vascular bed. *Am. J. Physiol.* **266(3 Pt 2),** H952-8 (1994).

[8] Lenasi, H. Assessment of Human Skin Microcirculation and Its Endothelial Function Using Laser Doppler Flowmetry. In *Medical Imaging* (ed. Erondu, O. F.). 271-296; 10.5772/27067 (IntechOpen, 2011).

[9] Roustit, M. & Cracowski, J. L. Assessment of endothelial and neurovascular function in human skin microcirculation. *Trends Pharmacol. Sci.* **34(7),** 373-84; 10.1016/j.tips.2013.05.007 (2013).

[10] Gaubert, M. L. *et al.* Endothelium-derived hyperpolarizing factor as an in vivo back-up mechanism in the cutaneous microcirculation in old mice. *J. Physiol.* **585(Pt 2),** 617-626 (2007).

[11] Cracowski, J. *et al.* Involvement of cytochrome epoxygenase metabolites in cutaneous postocclusive hyperemia in humans. *J. Appl. Physiol.* **114(2),** 245–251; 10.1152/japplphysiol.01085.2012 (2013).

[12] Brunt, V. E. & Minson, C. T. KCa channels and epoxyeicosatrienoic acids: major contributors to thermal hyperaemia in human skin. *J. Physiol.* **590(15),** 3523–3534; 10.1113/jphysiol.2012.236398 (2012).

[13] Smirni, S. *et al.* Application of cmOCT and continuous wavelet transform analysis to the assessment of skin microcirculation dynamics. *J. Biomed. Opt.* **23(7),** 076006; 10.1117/1.JBO.23.7.076006 (2018).

[14] Mayevsky, A. & Rogatsky, G. G. Mitochondrial function in vivo evaluated by NADH fluorescence: from animal models to human studies. *Am. J. Physiol. Cell Physiol.* **292(2),** C615-40 (2007).

[15] Drakaki, E. *et al.* Laser-induced fluorescence and reflectance spectroscopy for the discrimination of basal cell carcinoma from the surrounding normal skin tissue. *Skin Pharmacol. Physiol.* **22(3),** 158-65; 10.1159/000211912 (2009).

[16] Gillies, R., Zonios, G., Anderson, R. R. & Kollias, N. Fluorescence excitation spectroscopy provides information about human skin in vivo. *J. Invest. Dermatol.* **115(4),** 704-7 (2000).

[17] Heikal, A. A. Intracellular coenzymes as natural biomarkers for metabolic activities and mitochondrial anomalies. *Biomark. Med.* **4(2),** 241-63; 10.2217/bmm.10.1 (2010).

[18] Bolognia, J. L., Jorizzo, J. L. & Rapini, R. P. *Dermatology*. (Elsevier, 2003).

[19] Gupta, A., Avci, P., Dai, T., Huang, Y. Y. & Hamblin, M. R. Ultraviolet radiation in wound care: sterilization and stimulation. *Adv.. Wound Care (New Rochelle).* **2(8),** 422-437 (2013).

[20] Pasparakis, M., Haase, I. & Nestle, F. O. Mechanisms regulating skin immunity and inflammation. *Nat. Rev. Immunol.* 14(5), 289-301; 10.1038/nri3646 (2014).
